# Synergistic Effect of Poly(ethylenephosphoric Acid) and Cerium in Bone Substitute Composites on Tissue Response and Bone Remodeling

**DOI:** 10.3390/ijms262211113

**Published:** 2025-11-17

**Authors:** Victoria Besprozvannyh, Maria Ryndyk, Ilya Nifant’ev, Alexander Tavtorkin, Dmitry Gavrilov, Yulia Lukina, Leonid Bionyshev-Abramov, Natalya Serejnikova, Dmitriiy Smolentsev, Pavel Ivchenko

**Affiliations:** 1 A.V. Topchiev Institute of Petrochemical Synthesis RAS, Leninsky Pr. 29, 119991 Moscow, Russiampryndyk@edu.hse.ru (M.R.); tavtorkin@yandex.ru (A.T.); gavrosdm@gmail.com (D.G.); or inpv@org.chem.msu.ru (P.I.); 2 Faculty of Chemistry, National Research University Higher School of Economics, Myasnitskaya St. 20, 101100 Moscow, Russia; 3 Chemistry Department, M.V. Lomonosov Moscow State University, Leninskie Gory 1–3, 119991 Moscow, Russia; 4 N.N. Priorov National Medical Research Center for Traumatology and Orthopedics, Ministry of Health of the Russian Federation, Priorova St. 10, 127299 Moscow, Russia; lukina_rctu@mail.ru (Y.L.); sity-x@bk.ru (L.B.-A.); smolentsevdv@cito-priorov.ru (D.S.); 5 Faculty of Digital Technologies and Chemical Engineering, Mendeleev University of Chemical Technology of Russia, Miusskaya Sq. 9, 125047 Moscow, Russia; 6 Institute for Regenerative Medicine, Sechenov First Moscow State Medical University, Trubetskaya St. 8, 119991 Moscow, Russia

**Keywords:** biocompatibility, bioresorption, bone mineral substitute, carbonated apatite, cerium, composite, osteoconductivity, osteoinductivity, polyphosphodiesters, vascularization

## Abstract

To reduce the time of postoperative recovery and to prevent post-surgical complications, biocompatible synthetic materials with osteoconductive and osteoinductive properties are used as bone substitutes in large bone defect management. A simplified biomimetic approach to similar materials is based on the use of an inorganic filler, a polymer matrix, and a compatibilizer, mimicking the composition of the natural bone. Based on plate-like micro-sized carbonated hydroxyapatite (pCAp), we prepared compression-molded samples optionally containing an additional polyester component (poly(ε-caprolactone) PCL, poly(*L*-lactide) PLLA, or poly(*L*-methylglycolide) PLMG); syntheticblock copolymers comprising fragments of the corresponding polyester and poly(ethylene phosphoric acid) (PEPA) were also prepared and studied asa ‘two-in-one’ polymer matrix/compatibilizer. Bone regeneration experiments involving a three-month rat tibial defect model were conducted with 250–500 μm granules of the composites. Comparative studies of the introduction of the polyester-*b*-PEPA copolymer into composites revealed a positive effect, which manifests itself in accelerated bone regeneration, which further intensified for pCAp/PEPA-*b*-PLMG. The latter composite formulation was used to study the results of the introduction of cerium into the filler. One-month experiments with pCAp, CePO_4_-doped pCAp, and composites of these inorganic fillers with PEPA-*b*-PLMG were conducted. For the first time, a positive synergistic effect of the presence of cerium and PEPA in the composite, which appeared in substitution of the implant material by two-thirds of newly formed partly matured bone, was observed four weeks after surgery.

## 1. Introduction

The treatment of large segmental bone defects was and remains a topical scientific and practical problem in bone surgery and orthopedics [1]. For centuries, different materials have been studied as a substitutes formissing bone tissue [2]. The results of clinical trials revealed the ‘golden standard’ for bone replacement, autogenic graft, as well as the key problems and promising directions of the studies [3]. The development of bone substitutes (BSs), as a ‘synthetic, inorganic or biologically organic combinations which can be inserted for the treatment of a bone defect instead of autogenous or allogenous bone’ [4], is a challenging interdisciplinary problem at the intersection of chemistry, material science, biology, and medicine.

**Figure 1:**
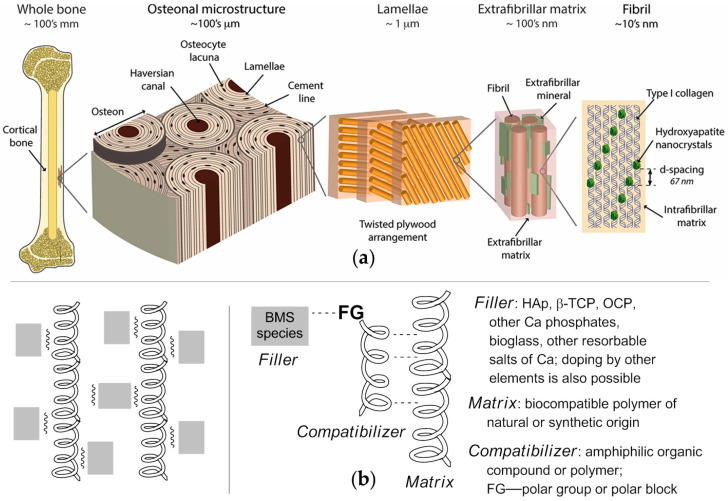
(**a**) The structure of cortical bone, adopted from [6], Creative Commons CC BY license (2016), Springer Nature; (**b**) three-component biomimetic approach to bone substitutes (BSs).

Our knowledge about the nature and structure of the human bone serves as an entry point for the development of BSs. Human cortical bone includes in its composition bone apatite (BA), organic components, and water; bone tissue (lamellae) comprises collagen fibrils, non-collagenous phosphorylated proteins (e.g.,osteopontin), and nano-sized plate-like crystallites of bone apatite [5,6,7,8]. The internal bone microstructure is organized into osteons containing a central Haversian canal for blood vessels and nerves (Figure 1a) [6].

The structure and composition of lamellae can be seen as a prototype for the development of BSs with the use of a biomimetic approach. With a similar approach, BSs may be considered as a composite material comprising bone mineral substitute (BMS) as a filler, an organic polymer as a matrix, and an amphiphilic organic compound performing the function of compatibilizer, similar to phosphorylated proteins (Figure 1b).

**Scheme 1:**
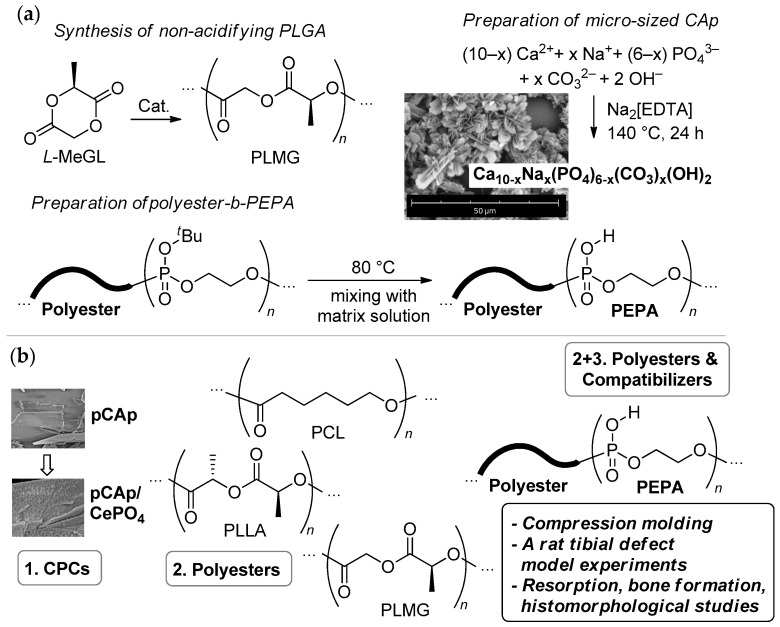
Experimental background (**a**) and the content (**b**) of the present work.

The principle of similarity may be involved in the development of each component. Osteoconductive inorganic materials, containing Ca^2+^, PO_4_^3–^, and other ‘biophilic’ ions, are seen as a prospective BMS (composite fillers, the first component of the composite) [9,10]. In many early works, FDA-approved [11] hydroxyapatite (HAp) was studied as a BMS [10,12,13]. Currently, there is a decreasing interest in using pure ‘stoichiometric’ HAp Ca_10_(PO_4_)_6_(OH)_2_ because it has reduced solubility, biodegradability, and fracture toughness [5,9,14]; however, HAp is still relevant in implant coating [15]. Other inorganic phases, e.g., FDA-approved [11] β-tricalcium phosphate (βTCP) [16], octacalcium phosphate [17], biphasic calcium phosphate [18,19,20], and different bioglasses [21], have been studied as a BMS. Carbonated apatite (CAp), close in composition to cortical bone apatite, also attracted researchers’ attention [5,9,22,23]. CAp scaffolds demonstrated exceptionally promising results in vivo, even when using partially amorphous material [24,25,26,27,28,29,30,31,32,33,34,35]. The problem of synthetic availability of perfectly shaped CAp material was resolved recently on micro-sized [36] (Scheme 1a) and nano-sized [37] levels. Comparative studies of CAp, HAp, and βTCP, conducted by Ishikawa and colleagues (hybrid dog mandible bone defect model) [25] and by our group (rat tibial model) [38], demonstrated higher rates of bone remodeling when using CAp instead of HAp and βTCP.

The polymer matrix (the second component in three-component composites) must possess biocompatibility and have quite high mechanical characteristics; in view of the further substitution by newly formed bone, the matrix has to be biodegradable at a suitable rate [13,39,40]. Non-toxic biodegradable poly(*L*-lactide) (PLLA) [41,42], poly(*L*-lactide-*co*-glycolide) (PLGA) [43,44], and, to a lesser extent, poly(ε-caprolactone) (PCL) [13,40,42], are attractive materials. However, PLLA has several drawbacks, such as low hydrophilicity, biodegradability and cell affinity, poor osteoconductivity, and insufficient strength and toughness for load-bearing applications [45]. The rates of biodegradability of PLGAs are notably higher than PLLA [44],which may cause an inflammatory response in surrounding tissues [46]. In [46], Meyer and colleagues showed that acidification during hydrolytic degradation of PLGA essentially depends on the copolymer microstructure and demonstrated the prospects of PLGA with an alternating comonomer sequence for biomedical use. In our recent study [47], we have shown that (co)polymers of *L*-methylglycolide (*L*-MeGL, Scheme 1a) represent ‘non-acidifying’ PLGAs. As a part of two-component composites with plate-like CAp (pCAp), these copolymers also demonstrated higher thermal and hydrolytic stability and better maintenance of the flexural strength in comparison with conventional PLGAs [48]. *L*-MeGL-based PLGAs have never been studied in bone remodelingin vivo.

The chemical binding with BMS and compatibility with the matrix (Figure 1b) provide dispersant and potentially hardening roles of the third component of the composite. Nature-like acidic –P(O)(OH)_2_ or >P(O)(OH) fragments are able to provide strong binding with the BMS surface [49], a positive effect of their application in BMS/polyester systems, manifested in deeper homogenization of the composites and marked increase in their strength, which was demonstrated recently by our group [50,51]. This became possible due to the use of a previously developed method of the synthesis of block copolymers [52] that form poly(ethylene phosphoric acid) (–OP(O)(OH)OCH_2_CH_2_–)*_n_* (PEPA) under mild conditions [53,54] (Scheme 1a). PEPA also holds promise for use in composites due to its osteoinductive properties revealed previously in vitro by our group [55] and by Iwasaki and colleagues [56] and its high mineral affinity [57,58]. Recently, during three-month in vivo experiments (a rat tibial defect model), we demonstrated that PEPA accelerates resorption of pCAp implants with partial formation of mature bone with Haversian systems [38]. The multifaceted function of PEPA in physiological processes is not limited by osteoinductivity. Iwasaki and colleagues showed that Na salt of PEPA easily penetrates into the macrophages [59] and promotes osteoblastic differentiation [60], which can not help but affect the processes of tissue healing and bone remodeling. Block copolymers of PEPA and polyesters de facto represent a combination of the polymer matrix (polyester) and compatibilizer (PEPA, covalently bonded with polyester).

Initial objectives of our study involved preparation and preliminary in vivo investigation of two-component pCAp/polyester and three-component pCAp/polyester-*b*-PEPA composites. Already, during the research, the academic interests of our group have been expanded by the studies of cerium-doped BMSs [61]. Interest in this topic was due to the major problem of the composite grafting—insufficient vascularization [3]. According to the results of numerous works [61,62,63,64,65], cerium-doped BMSs are able to promote vasculogenesis and angiogenesis during bone repair. In this way, the ultimate goals and objectives of our study also include the estimation of the effect of cerium in combination with three base components of the composite using rat tibial defect model experiments and necessary complementary investigations (Scheme 1b).

Below, we present the results of our study, during which, for the first time, comparative data on resorption and bone substitution of two-component (pCAp/polyester) and three-component (pCAp/polyester-*b*-PEPA) composites and on the impact of cerium on bone remodeling when using a rat tibial modelwere obtained.

## 2. Results and Discussion

### 2.1. Inorganic Filler

#### 2.1.1. Rationale for the Selection of the Filler

During our previous studies, we have developed hydrothermal methods for the synthesis of perfectly shaped microcrystalline CAp samples having different sizes and morphologies [36]. However, reproducibility of shapes and sizes of the crystallites was achieved in ongoing studies for hexagonal CAp and pCAp, among which pCAp showed better compatibility with polyesters [51]. In addition, the Ca^2+^-enriched surface in pCAp [66] increased its affinity for polyesters and PEPA. It is for these reasons that pCAp was selected as a BMS in the present work. The positive impact of PEPA on bone remodeling during implantation of pCAp was revealed previously [38], but the question about resorption of pCApas a component of the polymer composite required clarification.

Based on the literature data [61,62,63,64], we proposed that the cerium-containing inorganic phase might be a prospective additional component of the composite filler. It is well-known that introducing Ce^n+^ ions into the crystal lattice of pCAp (close to HAp) can be accompanied by substantial distortions of the lattice parameters. At high loadings of Ce, co-precipitation hydrothermal methods may result in changes of the crystallite size and morphology [67,68]. At high reaction temperatures, the CeO_2_ phase can also be formed [69]. With this in mind, Ce-containing phases were prepared by the treatment of pCAp by Ce(III) salt in aqueous solutionsby analogy with the method proposed recently for the synthesis of CePO_4_-decorated HAp nanofibers [70].

#### 2.1.2. Synthesis and Characterization of pCAp and Ce-Containing pCAp

pCAp was prepared according to the stoichiometry of type B CAp (Equation (1)) with the use of NaHCO_3_ (starting pH~6) in two stages: pre-treatment at 100 °C within 1 h with subsequent filtration and hydrothermal reaction at 140 °C within 24 h. The yield was 20% (10 g scale; for details, see Supplementary Materials).

(10 − x) Ca^2+^ + x Na^+^ + (6 − x) PO_4_^3–^ + x CO_3_^2–^ + 2 OH^–^→Ca_10−x_Na_x_(PO_4_)_6−x_(CO_3_)_x_(OH)_2_ (1)

**Figure 2:**
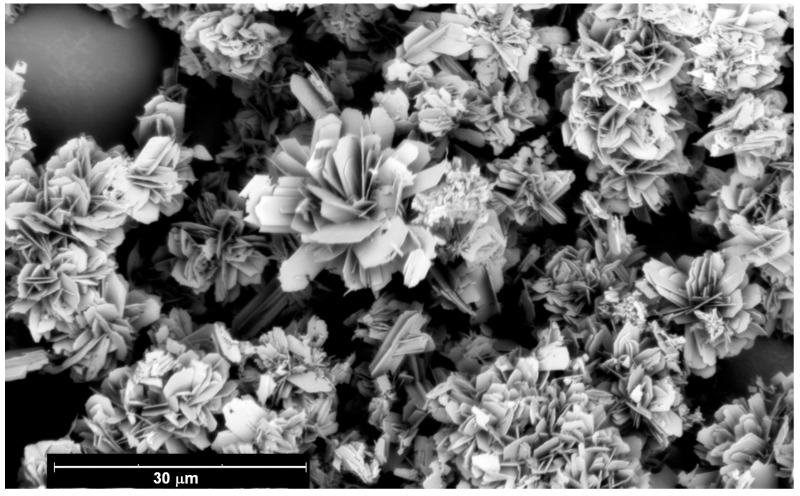
SEM image of plate-like carbonated apatite (pCAp). Acceleration voltage 15 kv.

X-ray diffraction studies confirmed B-type identity of pCAp;the content of CO_3_^2–^ anions was estimated using TGA and FT-IR spectroscopy (see Figures S1 and S2 in the Supplementary Materials), and the calculated [C]/[P] molar ratio was ~1:5 (the SEM/EDX method is hardly applicable for determination of carbon [71]; Figure S3). The content of [Na^+^], estimated by SEM/EDX, was ~10 mol.% from the content of [Ca^2+^], which roughly corresponds to Equation (1). The [Ca]/[P] molar ratio of ~1.7 in pCAp (EDX data) also corresponded to Equation (1) and was in the range of [Ca]/[P] ratios in natural bone (1.3–1.7) [72]. The control of the size and morphology of the pCAp sample (Figure 2) confirmed the reproducibility of the previously developed [51] method, which was used for the synthesis of the filler in the present study.

As mentioned in Section 2.1.1, preparation of Ce-doped pCAp was based on the precipitation of the CePO_4_ on the surface of crystallites in an aqueous medium. The use of this relatively new [70] approach to Ce-doped CPCs was driven by the desire to maintain the crystallite morphology of the filler. In our experiments, the weighted samples of pCAp were treated with an aqueous solution of a calculated amount of Ce(NO_3_)_3_; the reactions were conducted at 20 °C (8 h with stirring and subsequently 20 h of immersion). Initially, we proposed the formation of the phase Ca_9−*x*_Ce*_x_*Na(PO_4_)_5_CO_3_(OH)_2−*x*_O*_x_* via Ca^2+^→Ce^3+^ ion exchange. Two formulations of Ce-doped pCAp were prepared, with an anticipated content of 0.2 and 1 molar equivalents of Ce relative to the idealized molecular formula of type B CAp (Equation (1)) for *x* = 1. These phases are marked further as pCAp-Ce1 and pCAp-Ce2.

**Figure 3:**
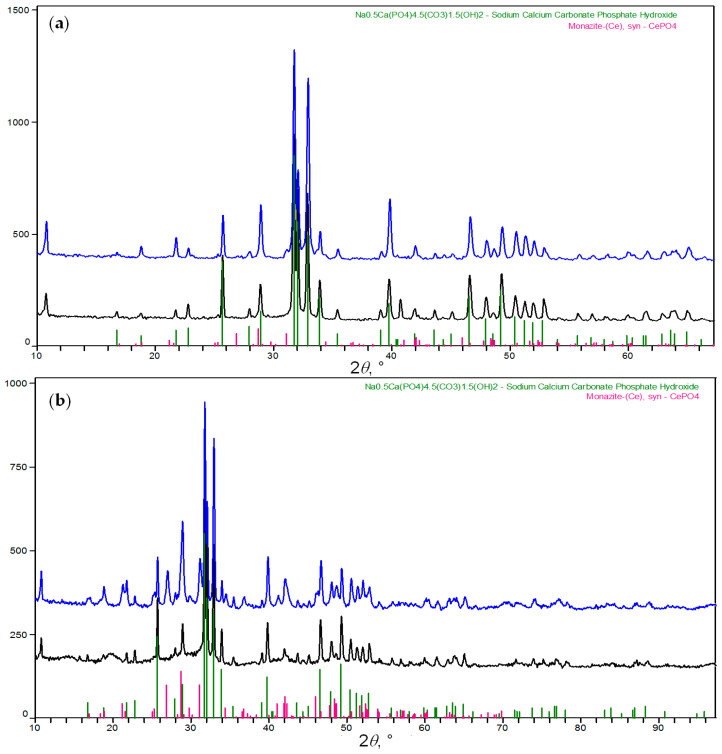
XRD traces of (**a**) pCAp-Ce1 and (**b**) pCAp-Ce2 (black and blue curves, before and after sintering at 600 °C, respectively) and comparative data for CAp and CePO_4_ (green and red lines, respectively; PDF^®^ database [74]). For pCAp-Ce2, the formation of CePO_4_ was detected.

However, in addition to Ca^2+^→Ce^3+^ ion replacement that had minimal impact on the HAp-type crystal structure of the filler at low degrees of ion substitution, XRD studies of pCAp-Ce1 and pCAp-Ce2 revealed the presence of a new crystalline phase, CePO_4_. As can be seen in Figure 3, the basic reflections of the CAp crystal phase remained virtually unchanged, but at the same time, clear structural markers of CePO_4_ were detected. The formation of this phase can be attributed to the high affinity of Ce^3+^ to PO_4_^3–^ and the stability of the CePO_4_ phase [70] that retains its chemical composition, even when heated in an oxygen atmosphere [73]. In this way, the reaction of pCAp with Ce^3+^ in the aqueous solution resulted in the formation of nano-sized crystallites of CePO_4_ at the surface of pCAp.

Before use in composite formulations, the samples of pCAp-Ce1 and pCAp-Ce2 were heated at 600 °C for the elimination of volatiles and guaranteed sterilization. In our previous studies, we have shown that the crystal structure of B-type pCAp does not visibly change at 650 °C [36], i.e., calcination at 600 °C could not change the identity of CAp. As can be seen in Figure 3, thermal treatment of Ce-doped pCAp also did not cause any noticeable changes in the crystal structure of the fillers.

**Figure 4:**
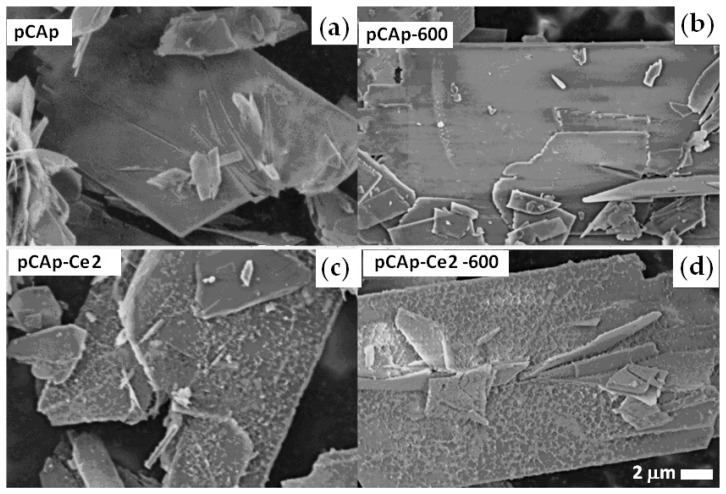
SEM images of pCAP (**a**); pCAp sintered at 600 °C (**b**); pCAp-Ce2 (**c**); pCAp-Ce2 sintered at 600 °C (**d**); acceleration voltage 5 kV. Base morphology of the crystallites remained unchanged; formation of the CePO_4_phase on the surface is clearly visible.

**Figure 5:**
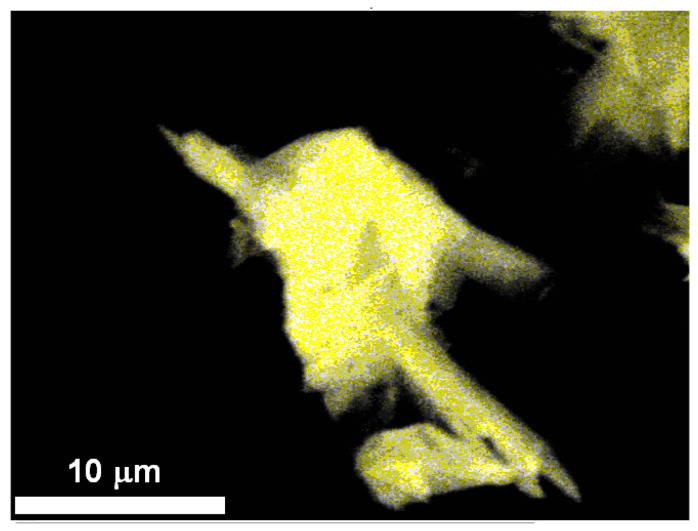
Multilayer SEM/EDX image of the sample ofthe pCAp-Ce2 crystallite. Ce atoms are evenly distributed at the surface.

SEM/EDX studies of the samples revealed the maintenance of the morphology of pCAp species during preparation of Ce-doped materials (Figure 4) and uniformity of the Ce distribution on the surface of the crystallites (Figure 5). When compared with a starting pCAp, the formation of an additional inorganic phase was observed on the surface. Before sintering, the Ce-containing phase was amorphous, and XRD patterns of CePO_4_ were observed in the case of sintered pCAp-Ce2 (appearance of the reflection at 2*θ* = 31°; Figure 3).

According to the results of multilayer SEM/EDX analysis, Ce atoms were uniformly distributed at the crystallite surface (Figure 5). The content of Ce in pCAp-Ce1 and pCAp-Ce2 was 2 and 8 wt.%, respectively. Comparative analysis of the Ce-containing samples using X-ray photoelectron spectroscopy (XPS) showed the major presence of Ce (III) (characteristic peak of Ce(IV) at 917 eV [75] was of low intensity), and the content of Ce was determined as 2.1 ± 0.2 and 8.4 ± 0.6 wt.% for pCAp-Ce1 and pCAp-Ce2, respectively.

### 2.2. Polymer Matrices and Compatibilizers

#### 2.2.1. Polymer Matrixes

**Scheme 2:**
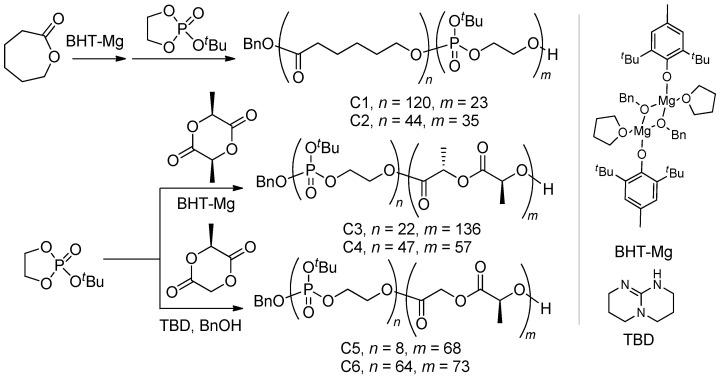
Synthesis of amphiphilic block copolymers C1–C6.

In our studies, we used commercial PCL (*M*_n_ = 88 kDa, *Ð*_M_ = 1.46) and PLLA (*M*_n_ = 107 kDa, *Ð*_M_ = 2.02). In light of increased actual interest in *L*-MeGL-based polymers [76,77,78], a homopolymer of *L*-MeGL (PLMG) was synthesized for its subsequent comparative studies in three-component composites. PLMG was prepared with the use of a [(BHT)Mg(μ-OBn)(THF)]_2_ single-component catalyst (BHT-Mg, Scheme 2), as described previously [79]. *M*_n_^NMR^ = 12.1 kDa (see Figure S6 in the Supplementary Materials), *M*_n_^SEC^ = 12.0 kDa, and *Ð*_M_ = 1.25.

#### 2.2.2. Block Copolymers of *^t^*BuOEP

Synthesis of block copolymers of 2-*tert*-butoxy-2-oxo-1,3,2-dioxaphospholane (*^t^*BuOEP) with a given comonomer composition is based on living ring-opening polymerization (ROP) and requires the use of an active and stable ROP catalyst. In our previous studies, the BHT-Mg catalyst (Scheme 2), developed for controlled ROP of different cyclic esters [80], demonstrated high reaction rates [52] and sufficient control of the degree of polymerization *DP*_n_ [53] when using *^t^*BuOEP as a monomer. The sequence of the introduction of cyclic esters and *^t^*BuOEP to copolymerization is determined by the nature of the active species, which have different chemical structures for lactones, lactides, and cyclic phosphates [81,82]. Block copolymers C1–C6, used in our study, were prepared according to Scheme 2. In the synthesis of C1 and C2, ε-caprolactone (εCL) was polymerized first; preparation of C1–C4 was conducted via polymerization of *L*-LA or *L*-MeGL, followed by the addition of *^t^*BuOEP. The values of *DP*_n_ for each comonomer were determined by end-group analysis of ^1^H NMR spectra of copolymers (see Section S2 in the Supplementary Materials).

### 2.3. Preparation of Composite Samples

#### 2.3.1. Preparation of the Composites

In our first study, devoted to the synthesis of PEPA and block copolymers of PEPA [53], quantitative formation of PEPA was observed after heating to 80 °C in protic solvents; separation and drying of copolymers were complicated by intermolecular polycondensation. It is for this reason that the preparation of the composites was based on the use of copolymers of *^t^*BuOEP. As was shown recently [51], the elimination of isobutylene proceeded with a quantitative formation of PEPA at the stage of the melt molding of the composites at high temperatures.

In the present study, the composites were prepared by the addition of calculated amounts (~85 wt%) of pCAp to the THF solution containing polyester or block copolymers C1–C4; copolymers C5 and C6 were dissolved in hexafluoroisopropanol (HFIP) due to the lower solubility of these copolymers in THF. The resulting mixtures were stirred at 90 °C for a hour. After elimination of the solvent, the residues were compressed with a load of 5 tons to tablets (1 mm thickness, 13 mm diameter) with subsequent crushing and sieving. The fraction of 250–500 μm was selected for implantation experiments.

The size of the composite particles was selected based on the results of previous studies of different BSs. For example, in critical-sized bone defect management, the following materials and optimal sizes of the particles (in brackets) were described: allograft (100–500 μm) [83], xenograft (250–100 μm) [84], calcium phosphate/silicate (250–500 μm) [85], HAp (355–500 μm) [86], CAp (425–600 μm) [87], and Herafill^®^ (CaSO_4_/CaCO_3_, 500–1000 μm) [88]. Additionally, 250–500 μm BMS particles were used in our recent work devoted to the comparison of different phosphate ceramics [38],setting the stage for comparison.

**Figure 6:**
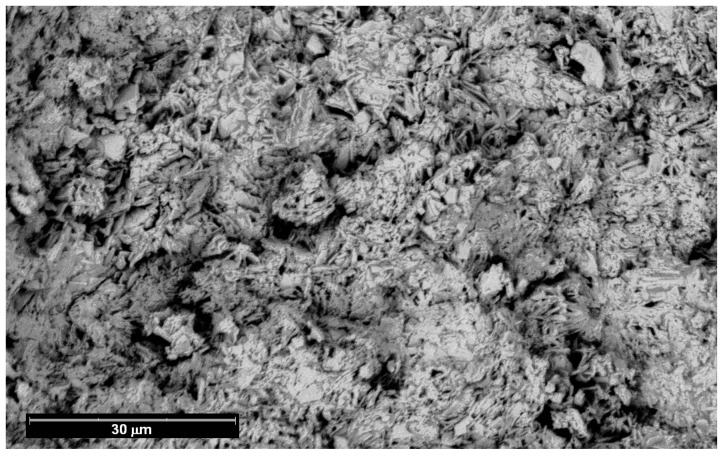
SEM image of the fracture surface of the composite C5/pCAp.

As can be seen in Figure 6, a substantial part of the pCAp species was subjected to only partial destruction during pressing. Maintaining the shape of the pCAp particles is desirable in view of their use in composites with a high content of the filler. It is not ultimately important for compressed samples prepared in this work; the mechanical properties of the composites are not particularly important in resorption experiments. However, our planned future studies of similar composites suggest that preparation of injection-molded articles and retention of the pCAp morphology are important for similar products.

**Figure 7:**
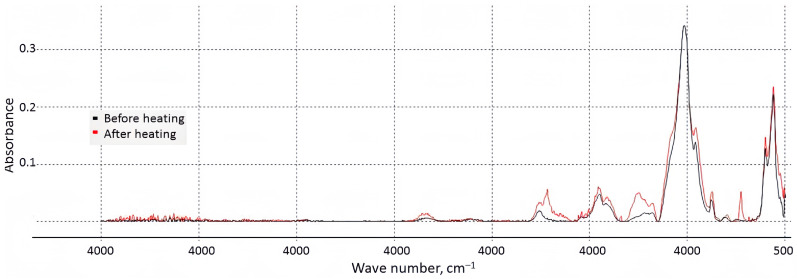
FT-IR spectra of C6/pCAp before and after heating at 90 °C (black and red lines, respectively).

The formation of PEPA at the first stage of the preparation of the composites was confirmed on the example of C5. As can be seen in Figure S20, after 1 h at 80 °C (external bath), the intensity of the bands at 2800–3000 cm^−1^ (C–H of *^t^*Bu groups) decreased substantially. Chemical bonding between PEPA fragments and pCAp was confirmed by FT-IR spectra of the composite remnants (after elimination of the solvent) before and after heating at 90 °C (Figure 7); the appearance of the spectral line at 1300 cm^–1^ corresponds to the interaction between the phosphate group and the Ca^2+^ ion [89].

#### 2.3.2. Composite Formulations in Light of the Objectives of the Study

Three different biodegradable polyesters, PCL, PLLA, and PLMG, were selected for our study. The composites with different ratios of pCAp and polyesters or C1–C6 copolymers were prepared to reveal the following patterns:

1. For two-component pCAp/polyester formulations, which of the three types of polyesters provides better biocompatibility and higher pCAp resorption?
2. For three-component pCAp/polyester-*b*-PEPA formulations,
  - Which of the three types of polyesters provides better biocompatibility, higher pCAp resorption, and more expressed new bone formation?
  - How does PEPA content in the copolymer influence these processes?
3. How does the presence of CePO_4_in pCAp influence composite resorption and bone remodeling?

The data on the composite formulations are presented in Table 1. The first series of experiments (3 months; see Section 2.4.2) was conducted to reveal issues 1 and 2. The second series of experiments (1 month; see Section 2.4.3) was devoted to the study of the effect of Ce. pCAp-Ce1 and pCAp-Ce2 were used as a filler for PLMG-based composites since PLMG appeared to be the most promising polyester matrix based on the results of the first series of experiments.

### 2.4. Bone Substitution and Regeneration Experiments

#### 2.4.1. Methodology and Description of the Experiments

The composite fraction of 250–500 μm size was selected for in vivo experiments using a rat tibial model of critical-sized bone defects (perforation of 6 × 3 mm on the outer of the proximal part of both tibiae). The duration of the trial was 3 months (series 1) or 30 days (series 2). The initial assessment of the resorption of the composite particles was performed with the use of computed tomography (micro-CT) throughout the experiments. In some cases, these studies revealed closure of the defect and resorption of the implant. Histomorphological studies were conducted after removal of the test animals from the experiment at day 90 or day 30.

#### 2.4.2. First Series of the Experiments: Effect of Polyester Type and PEPA

In the first series of experiments, we used the samples prepared from two-component (polyester/pCAp) and three-component (polyester-*b*-PEPA/pCAp) composites. For every comparative study of two selected composite formulations, a series of three laboratory animals was involved in the experiments. In all cases, micro-CT revealed positive dynamics of the closure of defects without the formation of exostoses and bone inflammation. However, PCL-, PLLA-, and PLMG-based materials demonstrated different rates of resorption.

In particular, for PCL-based composites, in all cases (PCL/pCAp, C1/pCAp, C2/pCAp), micro-CT revealed the presence of significant amounts of composite remnants three months after surgery. After implantation of PCL/pCAp, the defect was not closed completely (Figure S21a). In the case of PCL/pCAp (Figure S22), significantboneregeneration was observedin the bone lumen. Numerous implant remnants of the vacuole–fibrillar structure were surrounded by an indistinctly shaped immature trabecular bone, and small areas of fibroreticular tissue remained between the newly formed bone trabeculae. In the soft tissues, the remnants of the implant were surrounded by connective tissue capsules with a large number of multinucleated giant cells (MNGCs). For C1/pCAp and C2/pCAp (Figure S21c,d), micro-CT does not reveal the marked differences from PCL/pCAp. However, based on the results of histomorphological studies, the resorption of C2/pCAp appeared more pronounced (Figure S23). The composite remnants were surrounded by MNGCs; in part, the composite was replaced by immature trabecular bone. In this way, the presence of PEPA promoted the resorption of the material, but the high content of the MNGCs reflected moderate biocompatibility of the PCL-based composites.

For PLLA-based composites, micro-CT (Figure S24) did not reveal significant differences between the samples; the effect of PEPA was identified during histomorphological studies. In the case of PLLA/pCAp (Figure S25), good bone regeneration and weak composite resorption were observed; the entire lumen of the bone was filled with numerous large remnants of the implant with the vacuole–fibrillar structure (Figure S25c,d). In soft tissues, the remains of the implant were surrounded by connective tissue microcapsules, consisting mainly of MNGCs. A similar general picture was observed for C3/pCAp (Figure S26), but with a higher concentration of MNGCs that reflects the development of fibrosis around the remains of the implant in soft tissues. However, in the case of C4/pCAp, the overall picture was qualitatively different (see Figure S27). In the bone cavity, we observed not only a newly formed trabecular bone structure but also compact bone with a Haversian system. (Figure S27a–d). Few and small fragments of the implant inside the bone were observed, which indicated good resorption of C4/pCAp. In soft tissues, the rare remnants of the implant were surrounded by thin microcapsules, mainly containing macrophages (Figure S27d,e).

**Figure 8:**
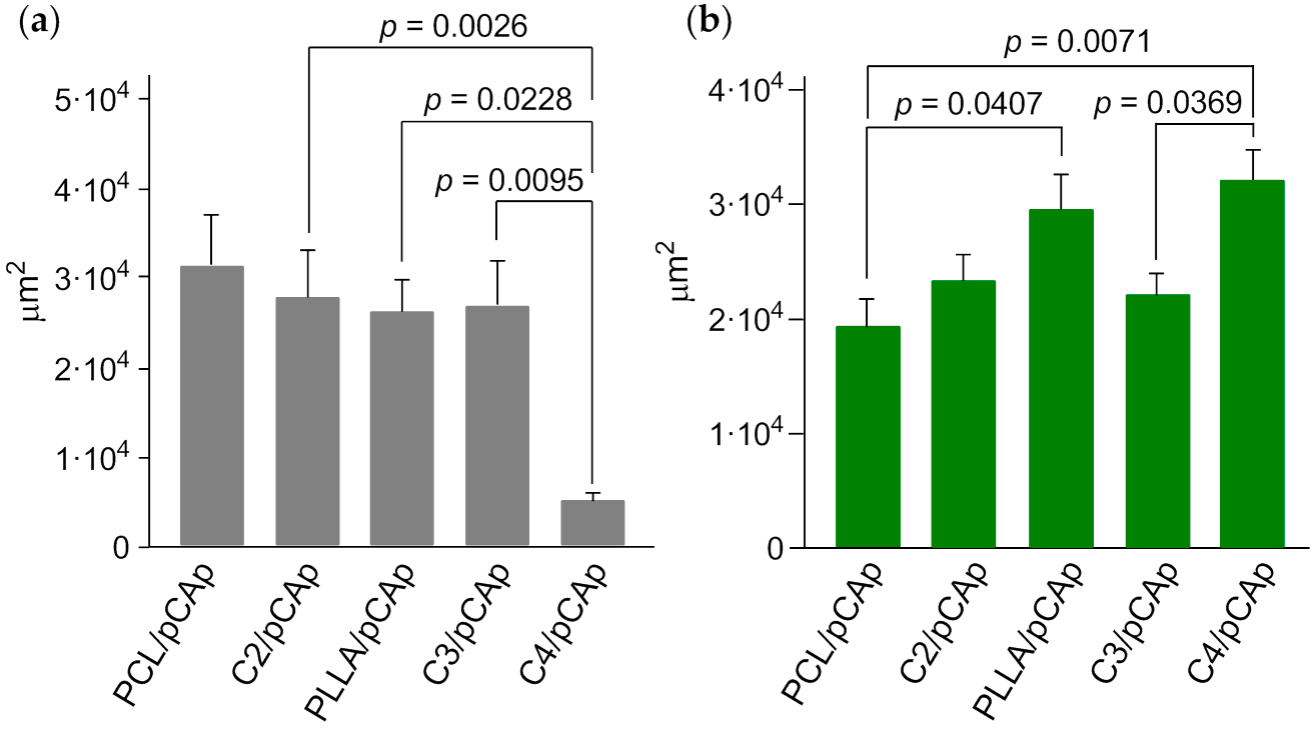
The areas of the composite remnants (**a**) and newly formed bone (**b**) (μm^2^/1 × 10^5^ μm^2^ bone surface area) after 3 months ofimplantation of the composite particles (250–500 μm). The error bars show SEM values; statistically significant differences (*p* ˂ 0.05) are shown.

The marked difference between composites, based on PCL and PLLA, is presented in Figure 8. The C4/pCAp copolymer falls out of the series. It can be attributed to the lower length of the PLLA fragment (*DP*_n_ = 57 in C4 vs. 136 in C3), and the higher content of PEPA also apparently played a part. An additional advantage of the C4/pCAp material over other samples was the absence of MNGCs after 3 months of implantation, which shows the better biocompatibility of this material. In this way, relatively high concentrations of PEPA promoted composite resorption and bone formation without visible negative side effects when using PLLA as a polymer matrix. PCL/pCAp materials seem unlikely to be applicable in orthopedics.

In the case of PLMG-based composites, in the absence of PEPA, we observed remnants of the implant after 3 months, but C5/pCAp and C6/pCAp were fully resorbed. PLMG seemed to be the most promising matrix among polyesters under study, but the three-month period of the first series of experiments turned out to be excessively long to identify the difference between C5 and C6. To reveal the potential of PLMG/PEPA in more detail and to study the effect of additional components of the composite (CePO_4_) on bone remodeling, the second series of shorter (1 month) experiments was carried out.

#### 2.4.3. Second Series of the Experiments: Effect of Cerium

As in the case of PCL- and PLLA-based composites, micro-CT studies did not reveal distinct differences between the samples, and histomorphological studies were supplemented by an estimation of the marker of osteogenesis (alkaline phosphatase activity) and a comparative evaluation of the rates of fibrosis and osteogenesis in the field of defect.

**Figure 9:**
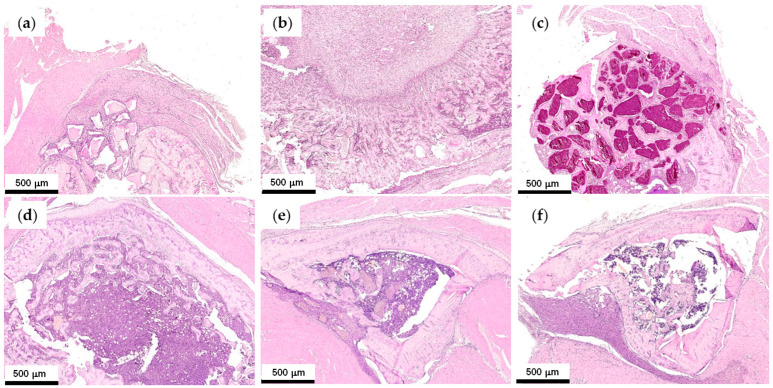
Hematoxylin/eosin stain images of the areas of implantation 1 month after the surgery: (**a**) pCAp; (**b**) pCAp-Ce2; (**c**) PLMG/pCAp; (**d**) C6/pCAp; (**e**) PLMG/pCAp-Ce2; (**f**) C6/pCAp-Ce2 (magnification 50×).

In our previous work [38], the positive effect of the addition of PEPA to pCAp was revealed during three-month experiments on the resorption of pCAp and PEPA/pCAp. In the present study, the time of the implantation was 1 month. Initially, we evaluated the difference in resorption of pCAp and Ce-doped pCAp; the sample pCAp-Ce2 with a higher content of CePO_4_ was selected. As expected, after 1 month, the resorption of pCAp was weak (Figure 9a), and osteoclasts were rare (Figure S28). pCAp granules were surrounded by cancellous bone tissue of varying degrees of maturity. Osteocytes were not numerous; the formation of Haversian canals and osteoblasts was detected in very rare cases. At the same time, MNGCs and macrophages actively participated in implant resorption in the soft tissues surrounding the defect area. Mallory staining revealed uniformly colored blue areas of immature reticulofibrous bone tissue (Figure S28). After 1 month, pCAp-Ce2 resorbed almost completely (Figure 9b). In the central part of the bone cavity, a loose network of reticulofibrous bone tissue was formed from connective tissue (Figure S29). Thin bone trabeculae with numerous osteoblasts were loosely located; the intertrabecular space was filled with bone marrow stroma. When moving from the center to the periphery of the bone cavity, the bone tissue gradually matured, the bone trabeculae thickened, the content of osteoblasts and osteocytes decreased, and the bone marrow cavities were filled with numerous blood vessels and the forming bone marrow. Single-forming Haversian canals with concentrically arranged bone plates of osteons were visible. When using Mallory staining, the areas of immature reticulofibrous bone tissue were uniformly colored blue (Figure S29). In this way, the presence of Ce promoted the resorption of pCAp, but this does not provide a rapid formation of the mature bone tissue.

In the case of PLMG/pCAp, the resorption was moderate (Figure 9c), and the defect area was filled with numerous fragments of implant surrounded by immature bone tissue with the signs of lamellar structure (Figure S30). Osteocytes, osteoclasts, and osteoblasts were rarely detected. The intertrabecular space was filled with red bone marrow. At the same time, MNGCs and macrophages actively participated in the resorption of the implant in the surrounding soft tissues with a formation of thin connective tissue microcapsules. Mallory staining revealed minor mature areas of the bone matrix (orange, Figure S30f). For C6/pCAp, minor amounts of the composite remnants and a higher degree of maturity of bone regeneration were observed (Figure S31). Immature spongy bone with a sign of a lamellar structure was formed by trabeculae with numerous osteoblasts on the surface, while in some areas, the trabeculae became thicker and denser, the bone matrix compactification increased, and the formation of Haversian canals was observed. In Mallory staining, the bony trabeculae were mostly uniformly colored blue. In this way, the presence of PEPA resulted in a significant increase in the rate of composite resorption, with a formation of immature bone tissue after 1 month. Therefore, the relative content of PEPA was of fundamental importance, as in the case of PEPA-*b*-PLLA.

**Figure 10:**
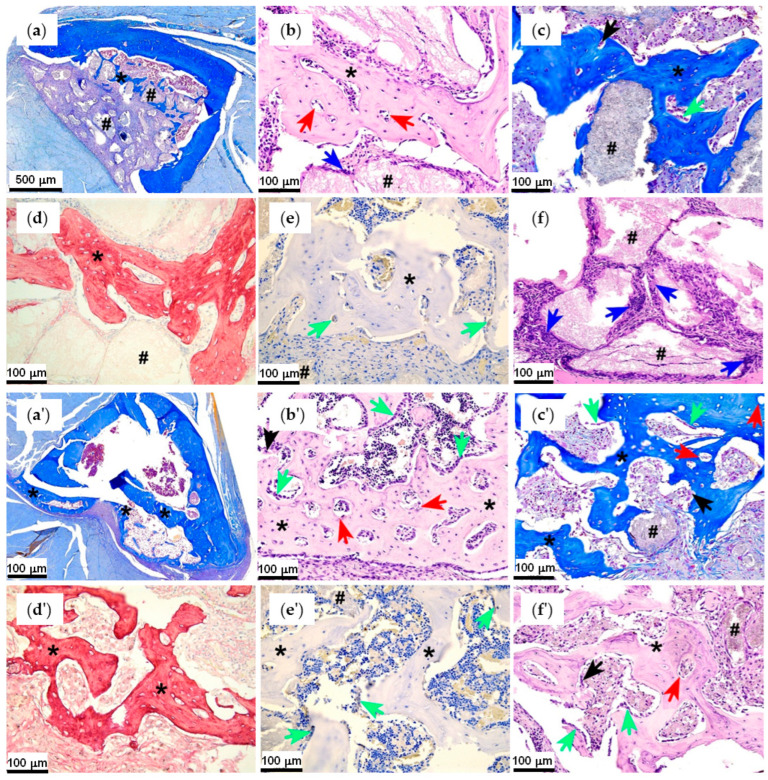
Comparative morphological analysis of the tissue response for C6/pCAp (**a**–**f**) and C6/pCAp-Ce2 (**a’**–**f’**). (**a**,**a’**) Common view of the defect area (Mallory staining, 50×), where implant remnants are clearly observed for C6/pCAp; (**b**–**f**,**b’**–**f’**) formation of new bone and cell environment (hematoxylin/eosin, Mallory, picrosirius red staining, 200×). Symbols: #—implant fragments; *—bone regenerate; the arrows indicate osteoblasts (green), osteoclasts (black), formation of Haversian canals (red), and MNGCs (blue).

The most interesting results were obtained for PLMG-based composites containing Ce-doped pCAp. When comparing PLMG/pCAp-Ce1 (Figure S32) and PLMG/pCAp-Ce2 (Figure S33), it can be concluded that an increase in the content of Ce resulted in an increase in the rate of resorption of the composite with a formation of more mature bone regenerate, though on a smaller scale. The addition of PEPA (C6/pCAp-Ce1) facilitated the rate of resorption with a formation of immature bone tissues (Figure S34). Finally, 1 month after the implantation of C6/pCAp-Ce2, we observed almost complete resorption of the composite (Figure S35) with a formation of immature lamellar bone tissue as a network of thickened trabeculae with numerous osteocytes, osteoblasts, and Haversian canals in the bone cavity. At the same time, the thickness of the trabeculae and the compactification of the bone matrix increased in the bone cavity at the border with the soft tissues, so that the immature bone acquired the signs of the compact bone. The difference between C6/pCAp and C6/pCAp-Ce2 is illustrated in Figure 10.

**Figure 11:**
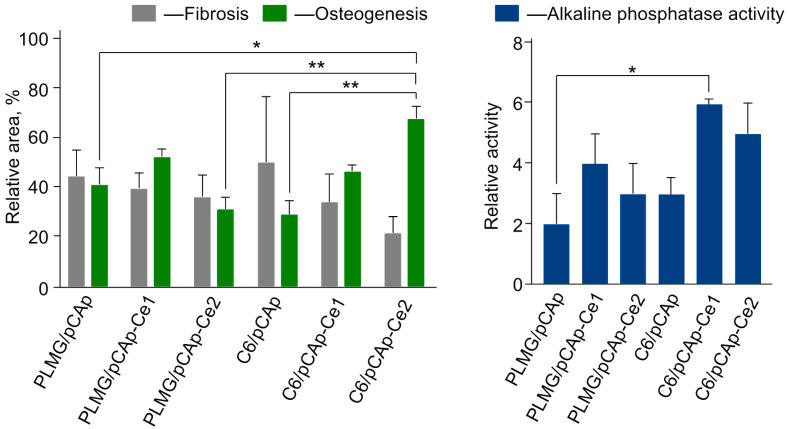
The relative areas of the fibrosis and osteogenesis (**left**) and relative activity of alkaline phosphatase (**right**) 1 month after the implantation of the composite particles (250–500 μm). The error bars show SEM values; *—*p* ˂ 0.05; **—*p* ˂ 0.01.

The main findings of the study are presented in Figure 11. As can be seen, the addition of relatively low amounts of CePO_4_ (pCAp-Ce1) facilitated new bone formation and decreased the fibrosis; the presence of PEPA had only modest effects on osteogenesis. However, at higher concentrations of Ce, we observed a marked synergistic effect of the simultaneous presence of CePO_4_ and PEPA in the composite, manifested in the formation of new bone with elements of mature structure in as little as one month after implantation.

The results of our studies showed that the presence of Ce facilitates vascularization in newly formed tissues. However, the relative number of Haversian canals in immature bone formed 1 month after the implantation of pCAp-Ce2 was relatively low;the implantation of C6/pCAp resulted in the formation of more mature bone tissue with a higher concentration of Haversian canals. The role of PEPA apparently consisted ofacceleration of the composite resorption and osteogenesis, and the effect of Ce is revealed in the presence of PEPA. Quantitative evaluation of vasculogenesis and angiogenesis requires longer experiments with the possible use of other animal models. A combination of PEPA and Ce-doped phosphate ceramics might be a hallmark of a ‘good’ composite for orthopedics, but the further development of similar composites and their introduction into medical practice requires additional comprehensive studies.

## 3. Materials and Methods

### 3.1. Solvents and Reagents

The solvents and reagents used in this work for the preparation of pCAp, BHT-Mg, monomers, and (co)polymers were supplied by Merck (Darmstadt, Germany). The solvents were refluxed and distilled over Na (toluene, diethyl ether (Et_2_O), tetrahydrofuran (THF), triethylamine (NEt_3_), *n*-pentane), CaH_2_ (CH_2_Cl_2_), and P_2_O_5_ (CH_3_CN). εCL and *N*,*N*-di(*n*-decyl)-*N*-methylamine were distilled in vacuo;*L*-LA was recrystallized from toluene and sublimed in vacuo. BHT-H (≥99%), di-*n*-butylmagnesium (1 M heptane solution), 1,5,7-triazabicyclo[4.4.0]dec-5-ene (TBD), acetic acid (AcOH), ethylene glycol, PCl_3_, *^t^*BuOH, CaCO_3_, NaHCO_3_, KH_2_PO_4_, Na_2_[EDTA]∙2H_2_O, Ca(NO_3_)_2_·4H_2_O, HFIP, and N-*p*-tolylbenzohydroxamic acid were used as purchased. For the preparation of the composites, commercial PCL (*M*_n_ = 88 kDa, *Ð*_M_ = 1.46, supplied by Shenzhen Bright China Industry, Shenzhen, China) and PLLA (*M*_n_ = 107 kDa, *Ð*_M_ = 2.02, supplied by FDplast, Moscow, Russia) were used. CDCl_3_ (D 99.8%) was supplied by Cambridge Isotope Laboratories, Inc. (Tewksbury, MA, USA).

BHT-Mg [80], *t*BuOEP [52,53], and *L*-MeGL [47,90] were synthesized as described previously (for details, see Section S1 in the Supplementary Materials).

### 3.2. Physico-Chemical Characterization

The Bruker AVANCE III HD 400 spectrometer (400 MHz, Bruker Corporation, Billerica, MA, USA) was used for registration of ^1^H and ^13^C NMR spectra;the chemical shifts are given relative to the residual peak of CDCl_3_ (7.26 ppm). FT-IR spectra (attenuated total reflection, ZnSe, 600–4000 cm^−1^) were recorded on an IFS 66v/S spectrometer with a DLaTGS detector (Bruker, Billerica, MA, USA). UV/vis spectra were recorded using a Varian Cary 100 spectrometer (Agilent Technologies, Santa Clara, CA, USA).

SEM images and energy-dispersive X-ray (EDX) analysis data were collected on a Phenom XL microscope (Thermo Fisher Scientific, Waltham, MA, USA). X-ray diffraction (XRD) patterns were obtained using a MiniFLEX 600 diffractometer (Rigaku Corp., Tokyo, Japan). Data were collected at 20 °C (2*θ* = 4.5–90°). The cell parameters were determined by full-profile fitting with the use of Bruker TOPAS 5 [91]. The PDF^®^ database [74] was used for the comparison of XRD patterns of the samples with reported data to determine the composition of the BMSs. XPS analysis was performed on the Thermo ARL Perform’x Sequential XFR instrument (Thermo Fisher Scientific).

SEC analyses of the (co)polymers were carried out on an Agilent PL-GPC 220 chromatograph (Agilent Technologies) in THF (1 mL∙min^–1^ at 40 °C, PLgel column, calibration to polystyrene standards).

### 3.3. Synthesis of Fillers

#### 3.3.1. Synthesis of pCAp

CaCO_3_ (50.0 g, 0.5 mol) and Na_2_[EDTA]∙2H_2_O (186 g, 0.5 mol) were dissolved in H_2_O (500 mL). The solution was filtered to a 1 L autoclave, pre-loaded with NaHCO_3_ (42.0 g, 0.5 mol) and KH_2_PO_4_ (40.83 g, 0.3 mol). After the addition of water (total volume 0.8 L), the autoclave was heated at 140 °C for 24 h. After cooling to 20 °C, the precipitate was separated by decantation, washed with H_2_O (2 × 150 mL), ^i^PrOH (2 × 100 mL), and pentane (2 × 50 mL), and then dried at 0.01 Torr. The yield was 9.45 g (20%).

#### 3.3.2. Synthesis of Cerium-Doped pCAp

The calculated amount of Ce(NO_3_)_3_∙6H_2_O (0.17 g, 0.40 mmol for the synthesis of CAp-Ce1, 0.87 g, 2.00 mmol for the synthesis of CAp-Ce2) was dissolved in H_2_O (10 mL). pCAp (2.00 g, ~2.0 mmol in accordance with Equation (1)) was added. The mixture was stirred at 20 °C for 8 h and stored for 20 h without stirring. The product was filtered off, washed withwater (5 × 40 mL), EtOH (2 × 40 mL), and pentane (2 × 50 mL), and dried at 0.01 Torr. The content of Ce was determined by UV-vis spectrophotometry of the complex with N-*p*-tolylbenzohydroxamic acid as described previously [92].

### 3.4. Synthesis of (Co)polymers

#### 3.4.1. PLMG

A solution of BHT-Mg (50 mg, 0.12 mmol) in CH_2_Cl_2_ (0.5 mL) was added to the solution of *L*-MeGL (1.56 g, 12 in) and CH_2_Cl_2_ (5.0 mL). After 12 h of stirring at 20 °C, AcOH (7.0 mg, 0.25 mmol) was added. The polymer was precipitated using a 10-fold volume excess of dry Et_2_O, with dissolution in CH_2_Cl_2_, and precipitation was repeated twice. The yield was 1.33 g (85%). ^1^H NMR (400 MHz, CDCl_3_, 20 °C) δ: 7.33 (m, 5H, aromatic ring); 5.22 (m, 93H, –C*H*<); 4.87—4.60 (group of d, 186 H, –C*H*_2_–); 1.56 (d, ^3^*J* = 7.1 Hz, 280H, –C*H*_3_). ^13^C NMR (101 MHz, CDCl_3_, 20 *°*C) δ: 169.46; 166.53; 69.21; 60.89; 16.80.

#### 3.4.2. εCL Copolymers C1 and C2

A solution of BHT-Mg (50 mg, 0.12 mmol) in CH_2_Cl_2_ (0.5 mL) was added to the solution of εCL (1.08 g, 9.5 mmol) in CH_2_Cl_2_ (4.0 mL). After 90 min of stirring at 20 °C, *^t^*BuOEP (0.43 g, 2.4 mmol) was added. Copolymerization was terminated after 12 h at 20 °C by the addition of AcOH (7.0 mg, 0.25 mmol). The reaction mixture was poured into Et_2_O (50 mL), the copolymer was filtered off and dissolved in CH_2_Cl_2_ (4 mL), and the procedure was repeated three times. The yield was 1.16 g (74%). ^1^H NMR (400 MHz, CDCl_3_, 20 *°*C) δ: 7.32 (m, 5H, *Ph*CH_2_); 5.08 (s, 2H, PhC*H*_2_); 4.16 (m, 90H, POC*H*_2_); 4.03 (t, ^3^*J* = 6.8 Hz, 240H); 2.28 (t, ^3^*J* = 7.4 Hz, 240H); 1.62 (m, 480H); 1.48 (s, 203H, *^t^*Bu); 1.35 (m, 240H). ^31^P{^1^H} NMR (162 MHz, CDCl_3_, 20 *°*C): δ –5.42 (1P); −5.69 (22P). The composition of C1 was determined as BnO(εCL)_120_-*b*-(*^t^*BuOEP)_23_ by the analysis of ^1^H and ^31^P NMR spectra (Figures S6 and S7 in the Supplementary Materials).

Copolymer C2 was obtained by the same method using εCL (1.08 g, 9.5 mmol), BHT-Mg (100 mg, 0.24 mmol), and *^t^*BuOEP (1.7 g, 9.5 mmol). The yield was 2.2 g (80%). The composition of C2 was determined as BnO(εCL)_44_-*b*-(*^t^*BuOEP)_35_ by the analysis of ^1^H and ^31^P NMR spectra (Figures S8 and S9 in the Supplementary Materials).

#### 3.4.3. *L*-LA Copolymers C3 and C4

A solution of BHT-Mg (0.1 g, 0.24 mmol) in CH_2_Cl_2_ (1.0 mL) was added to the solution of *^t^*BuOEP (0.85 g, 4.7 mmol) in CH_2_Cl_2_ (3.0 mL). After 2 h of stirring at 20 °C, a solution of *L*-LA (2.73 g, 19 mmol) in CH_2_Cl_2_ (4.5 mL) was added. Copolymerization was terminated after 16 h at 20 °C by the addition of AcOH (14 mg, 0.5 mmol). The copolymer was separated similarly to C1. The yield was 2.52 g (69%). ^1^H NMR (400 MHz, CDCl_3_, 20 °C) δ: 7.37 (m, 5H, *Ph*CH_2_); 5.16 (q, ^3^*J* = 7.1 Hz, 274H, C*H*CH_3_); 5.04 (d, 2H, PhC*H*_2_); 4.18 (m, 85H, POC*H*_2_); 1.57 (d, ^3^*J* = 7.1 Hz, 812H, CHC*H*_3_); 1.50 (s, 208H). ^31^P{^1^H} NMR (162 MHz, CDCl_3_, 20 *°*C): δ—5.48 (1P); −5.65 (21P). The composition of C3 was determined as BnO(*^t^*BuOEP)_22_-*b*-(*L*-LA)_136_ by the analysis of ^1^H and ^31^P NMR spectra (Figures S10 and S11 in the Supplementary Materials).

Copolymer C4 was obtained by the same method using *^t^*BuOEP (0.85 g, 4.7 mmol), BHT-Mg (50 mg, 0.12 mmol), and *L*-LA (0.68 g, 4.7 mmol). The yield was 1.1 g (72%). The composition of C4 was determined as BnO(*^t^*BuOEP)_47_-*b*-(*L*-LA)_57_ by the analysis of ^1^H and ^31^P NMR spectra (Figures S12 and S13 in the Supplementary Materials).

#### 3.4.4. *L*-MeGL Copolymers C5 and C6

A solution of TBD (20 mg, 0.14mmol) and BnOH (15 mg, 0.14 mmol) in CH_2_Cl_2_ (1.0 mL) was added to the solution of *^t^*BuOEP (0.52 g, 2.9 mmol) in CH_2_Cl_2_ (1.5 mL). After 1.5 h of stirring at 20 °C, a solution of *L*-MeGL (1.5 g, 11.5mmol) in CH_2_Cl_2_ (3.0 mL) was added. Copolymerization was terminated after 3 h at 20 °C by the addition of AcOH (14 mg, 0.5 mmol). The copolymer was separated similarly to C1. The yield was 1.32 g (65%). ^1^H NMR (400 MHz, CDCl_3_, 20 *°*C) δ: 7.37 (m, 5H, *Ph*CH_2_); 5.25–5.14 (mm, 68H, C*H*CH_3_); 5.04 (d, 2H, PhC*H*_2_); 4.89–4.60 (mm, CH_2_, 136H); 4.18 (m, 30H, POC*H*_2_); 1.57 (d, ^3^*J* = 7.1 Hz, CHC*H*_3_); 1.49 (s) {total 286H}. ^31^P{^1^H} NMR (162 MHz, CDCl_3_, 20 °C): δ—5.50 (1P); −5.66 (7P). The composition of C5 was determined as BnO(*^t^*BuOEP)_8_-*b*-(*L*-MeGL)_68_ by the analysis of ^1^H and ^31^P NMR spectra (Figures S14 and S15 in the Supplementary Materials).

Copolymer C6 was obtained by the same method using *^t^*BuOEP (1.0 g, 5.6 mmol), TBD (16 mg, 0.11 mmol), and *L*-MeGL (0.72 g, 5.6 mmol). The yield was 1.43 g (84%). The composition of C6 was determined as BnO(*^t^*BuOEP)_64_-*b*-(*L*-MeGL)_73_ by the analysis of ^1^H and ^31^P NMR spectra (Figures S16 and S17 in the Supplementary Materials).

### 3.5. Preparation of the Composite Samples

#### 3.5.1. PCL-, PLLA-,and PLMG-Based Composites

In a tightly closed round-bottom glass vial, calculated amounts of the polyester or *^t^*BuOEP blockcopolymer were dissolved in THF (10 μL/mg), and a calculated amount of pCAp (see Table 1) was added with stirring (180–240 rpm, oval magnetic stirring bar). The mixture was heated for 1 h (120 rpm; the temperature of the external bath was 90 °C), cooled to room temperature, and excess pressure was dissipated safely. The suspension was evaporated under reduced pressure; the residue was ground, placed into a mold (1 × 13 mm), and pressed with 5 t loading.

#### 3.5.2. C5- and C6-Based Composites

The composite samples in tablet form were prepared as described in Section 3.5.1, except that the calculated amount of C5 or C6 (see Table 1) was dissolved in HFIP (10 μL/mg).

### 3.6. In Vivo Experiments

#### 3.6.1. Animals

Eight-week-old male Wistar rats (Shemyakin and Ovchinnikov Institute of Bioorganic Chemistry RAS, Pushchino, Russia) were used in the study. All animal procedures were performed according to common related manuals for the protection of animals used for scientific purposes (the guide for care and use of laboratory animals [93], EC Directive 2010/63/EU) and the Standards of Good Laboratory Practice (Order No. 199n of the Ministry of Health of the Russian Federation, 1 April 2016). The animals were adapted to laboratory conditions (23 °C, 12 h light/12 h dark, 50% humidity, unlimited access to food and water) within 14 days prior to the experiments.

#### 3.6.2. Bone Repairing Studies

The studies of biocompatibility and resorption of the composites were carried out on 11 series of 3 male Wistar rats (250–300 g), with the use of a tibial model of a critical-sized bone defect (3–4 × 6 mm perforation). The material was implanted directly and tamped into the bone marrow cavity with a minimal excess outside the bone surface. The experiments lasted for 3 months (first series) or 1 month (second series), after which the rats were sacrificed in a CO_2_ chamber.

Microcomputer tomography was performed with a Prodis. Compact 2430TC desktop microtomography system (Prodis.NTD, Moscow, Russia) at a voltage of 100 kV and a current of 100 μA, with a 0.3 mm CU filter. Sliceswere reconstructed using Prodis. Electro 1.3.20240214 software; visualization was performed using Prodis VolAn 0.3.4 software. The spatial resolution when scanning the entire rat (intravitalmicro-CT) was about 30 micronvoxels; when scanning autopsy material, it was about 9 micronvoxels.

#### 3.6.3. Morphological Studies

The tissue samples were fixed in a 10% formalin-buffered solution, immersed in a SoftiDek EDTA-based decalcifying solution (Biovitrum, St. Petersburg, Russia) for 7 days, and processed routinely for paraffin histology. Histological sections (a thickness of 4 μm) were prepared using a Leica RM 2125 RTS microtome (Leica Microsystems, Wetzlar, Germany) and stained withhematoxylin/eosin, Mallory’s trichrome (Biovitrum, St. Petersburg, Russia), Picrosirius Red (Abcam, Cambridge, UK), and immunohistochemically for alkaline phosphatase (primary rabbit polyclonal antibodies DF6225, Affinity Biosciences, Blackburn North, Australia; secondary goat anti-rabbit antibodies 205718, Abcam). All staining procedures followed standard histological protocols [94,95,96,97]. All histological sections were examined under bright-field and polarization microscopies in a Leica DM 4000 B LED microscope with a Leica DFC 7000 T. 8 camera (Leica Microsystems GmbH, Wetzlar, Germany). Digital imaging of the slides was performed using a Hamamatsu NanoZoomer S20MD high-resolution slide scanner (Hamamatsu Photonics, Hamamatsu, Shizuoka, Japan) for morphometric analysis. Using the NDP.view2 program (Hamamatsu Photonics), the following histological images per 1 mm^2^ of section area were evaluated: the relative area of bone regenerates, the relative area of implant fragments, the percentage of the area occupied by bone regenerates with implant fragments, the percentage of the area occupied by fibrous tissue with implant fragments, and the ratio of osteogenesis and fibrosis processes. The results of immunohistochemistry were assessed using a semi-quantitative method based on the total score. Six histological sections were randomly selected for each sample; micrographs were made with 10 fields of view for all sections.

### 3.7. Statistical Analysis

Statistical analysis was performed using the GraphPad Prism program (v. 10.00 for Windows, GraphPad Software, Inc., San Diego, CA, USA). The normality of the distribution was determined using the Shapiro–Wilk test (α > 0.05). The significance of the differences was assessed using a one-factor ANOVA with Tukey’s multiple comparison test for quantitative data and the Kruskal–Wallis test followed by Dunn’s multiple comparison test for scoring. The results of the statistical analysis were presented as a histogram of averages and errors of the mean or median values and a 95% confidence interval; the values of *p* < 0.05 were considered statistically significant.

## 4. Conclusions

For the first time, three-component composites, comprising plate-like micro-sized carbonated apatite pCAp and synthetic polyesters of different natures (PCL, PLLA, or poly(*L*-methylglycolide) PLMG), or pCAp and block copolymers of the corresponding polyesters with poly(ethylenephosphoric acid) (PEPA) C1–C6, were prepared by compression molding. A total of 250–500 μm composite particles were applied in comparative studies of bone repair with the use of a rat tibial defect model. Three months after surgery, PLLA- and PLMG-based composites C4/pCAp and C6/pCAp with higher PEPA content showed facilitated resorption, and new tissues with signs of mature bone were formed.

The effect of the presence of CePO_4_ in composite fillers was studied in 1-month experiments using the same model. For PLMG-based composites, additional introduction of cerium resulted in further improvement of the composite characteristics. Higher biocompatibility of Ce-containing implants was confirmed by the absence of multinucleated giant cells (MNGCs); newly formed lamellar bone had signs of compact bone with the Haversian system. In the presence of both Ce and PEPA, the highest rates of composite resorption and new bone formation were observed. After 1 month, the relative area of bone regeneration exceeded 60%.

The size and morphology of pCAp and pCAp/CePO_4_ are close, and the latter inorganic phase can be viewed as a prospective filler for the preparation of biomimetic three-component composites, based on BMS, polyester, and a PEPA-containing compatibilizer. PLMG represents a new and poorly studied polymer matrix for composite formulations, and further development of *L*-methylglycolide-based materials for bone surgery and orthopedics is quite promising.

The results of our study were obtained in the framework of the small rodents’ critical size defect model with the use of a standard size defect [98] in rat femoral bone. This method has moderate translational potential for use in bone surgery and orthopedics [99]. The size and composition of the BMSs developed and studied in this work are still far from the properties of the materials suitable for clinical trials. However, in our opinion, the osteoinductivity of PEPA and the complex effect of PEPA and Ce phases, accelerating BMS resorption and bone formation, have a high potential for transferability to medical practice.

## Figures and Tables

**Table 1:** Formulations of the composites prepared and studied in this work.

Composite	(Co)polymer, wt.%	PEPA, mol.% in Copolymer	Animals Series ^1^
First series of experiments (3 months)			
PCL/pCAp	15	0	A, B
C1/pCAp	16	16	A
C2/pCAp	15	44	B
PLLA/pCAp	15	0	C
C3/pCAp	16	14	C, D
C4/pCAp	16	45	D
PLMG/pCAp	15	0	E, F
C5/pCAp	16	11	E
C6/pCAp	15	47	F
Second series of experiments (1 month)			
pCAp	0	0	G
pCAp-Ce2	0	0	G
PLMG/pCAp	15	0	H
C5/pCAp	16	11	H
C6/pCAp	15	47	I
PLMG/pCAp-Ce1	15	0	I
PLMG/pCAp-Ce2	15	0	J
C6/pCAp-Ce1	16	47	K
C6/pCAp-Ce2	16	47	J, K

^1^ Three animals in a series.

## Data Availability

The raw data supporting the conclusions of this article will be made available by the authors upon request.

## Supplementary Materials

The following supporting information can be downloaded at https://www.mdpi.com/article/10.3390/ijms262211113/s1.

## Abbreviations

The following abbreviations are used in this manuscript:

BA	Bone apatite
BHT	Butylated hydroxytoluene, 2,6-di-*tert*-butyl-4-methylphenol
BMS	Bone mineral substitute
BS	Bone substitute
CPCs	Calcium phosphate ceramics
CT	Computed tomography
*DP*_n_	Degree of polymerization
EDTA	Ethylenediamine tetraacetate
HAp	Hydroxyapatite
HFIP	Hexafluoroisopropanol
*L*-LA	*L*-lactide
*L*-MeGL	*L*-methylglycolide
MNGCs	Multinuclear giant cells
pCAp	Plate-like carbonated apatite
PCL	Poly(ε-caprolactone)
PEPA	Poly(ethylenephosphoric acid)
PLLA	Poly(*L*-lactide)
PLMG	Poly(*L*-methylglycolide)
SEC	Size exclusion chromatography
SEM/EDX	Scanning electron microscopy/energy-dispersive X-ray spectroscopy
βTCP	β-tricalcium phosphate
THF	Tetrahydrofuran
XRD	X-ray diffraction
