# Supplementary material for: Synergistic Effect of Poly(ethylenephosphoric Acid) and Cerium in Bone Substitute Composites on Tissue Response and Bone Remodeling
Source: Int J Mol Sci. 2025 Nov 17;26(22):11113. doi: 10.3390/ijms262211113 (PMC12653750; doi:10.3390/ijms262211113)
Supplement: Supplementary file 1 [file ijms-26-11113-s001.zip › ijms-3952059_Supplementary_R3.pdf]

Synergistic Effect of Poly(Ethylenephosphoric Acid) and Cerium in Bone Substitute Composites on Tissue Response and Bone Remodeling

Victoria Besprozvannyh[1,2], Maria Ryndyk[1,2], Ilya Nifant'ev [1,2,3,*], Alexander Tavtorkin [1], Dmitry Gavrilov [1,3], Yulia Lukina [4,5], Leonid Bionyshev-Abramov [4], Natalya Serejnikova [4,6], Dmitriiy Smolentsev [4], Pavel Ivchenko [1,3]

[1] A.V. Topchiev Institute of Petrochemical Synthesis RAS, Leninsky Pr. 29, 119991 Moscow, Russia; inif@ips.ac.ru (I.N.); tavtorkin@yandex.ru (A.T.); phpasha1@yandex.ru (P.I.); gavrosdm@gmail.com (D.G.).
[2] Faculty of Chemistry, National Research University Higher School of Economics, Myasnitskaya st. 20, 101100 Moscow, Russia; mpryndyk@edu.hse.ru (M.R.); vkbesprozvannykh@edu.hse.ru (V.B.)
[3] Chemistry Department, M.V. Lomonosov Moscow State University, Leninskie Gory 1–3, 119991 Moscow, Russia; inif@org.chem.msu.ru (I.N.); inpv@org.chem.msu.ru (P.I.).
[4] N.N. Priorov National Medical Research Center for Traumatology and Orthopedics, Ministry of Health of the Russian Federation, Priorova st. 10, 127299 Moscow, Russia; lukina_rctu@mail.ru (Y.L.); sity-x@bk.ru (L. B.-A.); natalia.serj@yandex.ru (N.S.); SmolentsevDV@cito-priorov.ru (D.S.)
[5] Faculty of Digital Technologies and Chemical Engineering, Mendeleev University of Chemical Technology of Russia, Miusskaya sq. 9, 125047 Moscow, Russia.
[6] Institute for Regenerative Medicine, Sechenov First Moscow State Medical University, Trubetskaya st. 8, 119991 Moscow, Russia.
* Correspondence: inif@ips.ac.ru; inif@org.chem.msu.ru

Supplementary Information

S1. Carbonated Apatite and Cerium-Doped Carbonated Apatite S2
S2. Synthesis of Copolymers S4
S3. Preparation and Laboratory Testing of the Composites S13
S4. Comparative *in vivo* Studies of the Composites S13
References S25

S1. Carbonated Apatite and Cerium-Doped Carbonated Apatite

S1.1. XRD analysis of BMS

During the study of pCAp sample, cell parameters were determined by Pawley refinement. Experimental (blue) and calculated (red) diffractograms, difference curves (gray) and impurity peaks (vertical lines) are presented in Figure S1. XRD pattern of pCAp confirms its Type B CAp identity and belonging to HAp family of CPCs.

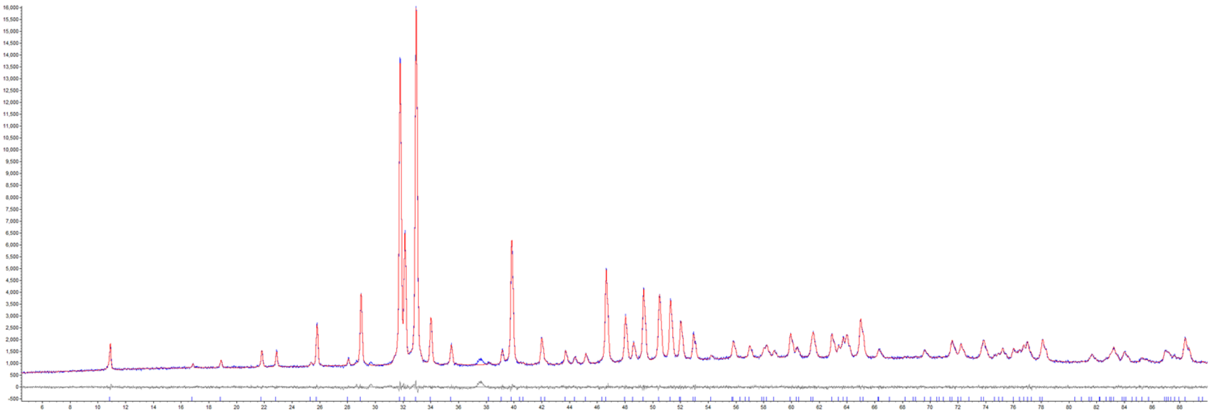

Figure S1. Experimental (blue) and calculated (red) XRD patterns of pCAp.

S1.2. FT-IR spectra of BMS

The formation of the Type B CAp comes from the substitution of PO_4^{3-} and OH^- by CO_3^{2-} in HAp that can be detected by FT-IR spectroscopy. The characteristic bands of carbonate group are observed in the spectral regions 1400–1600 cm^{-1} (ν_3; asymmetric stretching vibration) and 870–880 cm^{-1} (ν_2; out-of-plane bending vibration). The carbonate ν_3 band at 1465 cm^{-1} is a IR signature of Type B CAp.

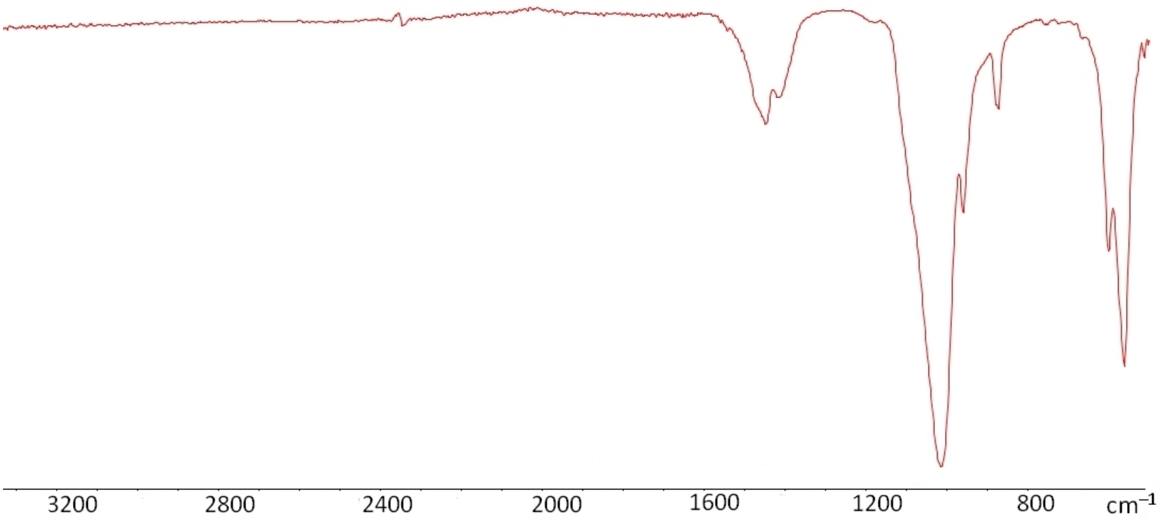

Figure S2. FT-IR spectra of pCAp.

The Figure S3 illustrates limited applicability of the SEM/EDX for qualitative determination of 'light' elements (C, O).

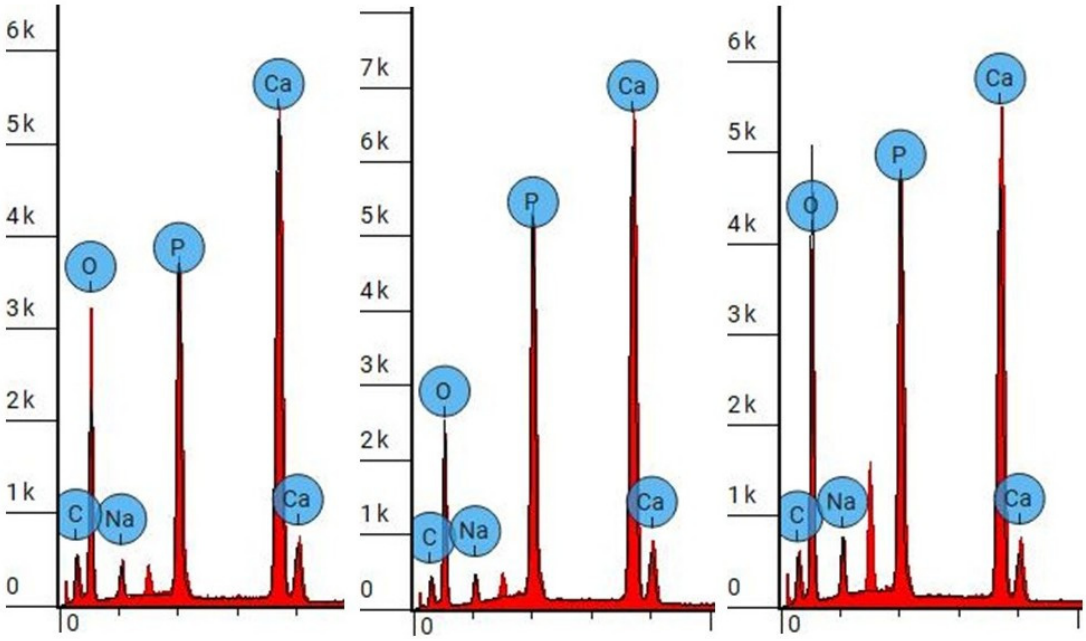

Figure S3. EDX data for pCAp.

S2. Synthesis of Copolymers

S2.1. Synthesis and Purification of the Monomers

2-(*Tert*-butoxy)-1,3,2-dioxaphospholane 2-oxide (*tert*-butylethylene phosphate, ^{t}BuOEP)

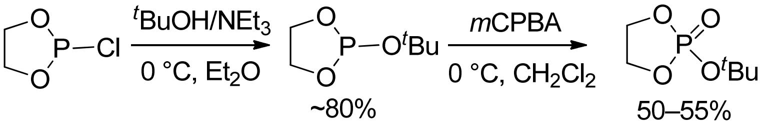

The synthesis of ^{t}BuOEP was conducted as described previously [1,2], the product was purified by distillation in the presence of *N*,*N*-di(*n*-decyl)-*N*-methylamine (5 wt%) [3]. The typical yields of polymerization product grade (Figure S1) were 50–55%.

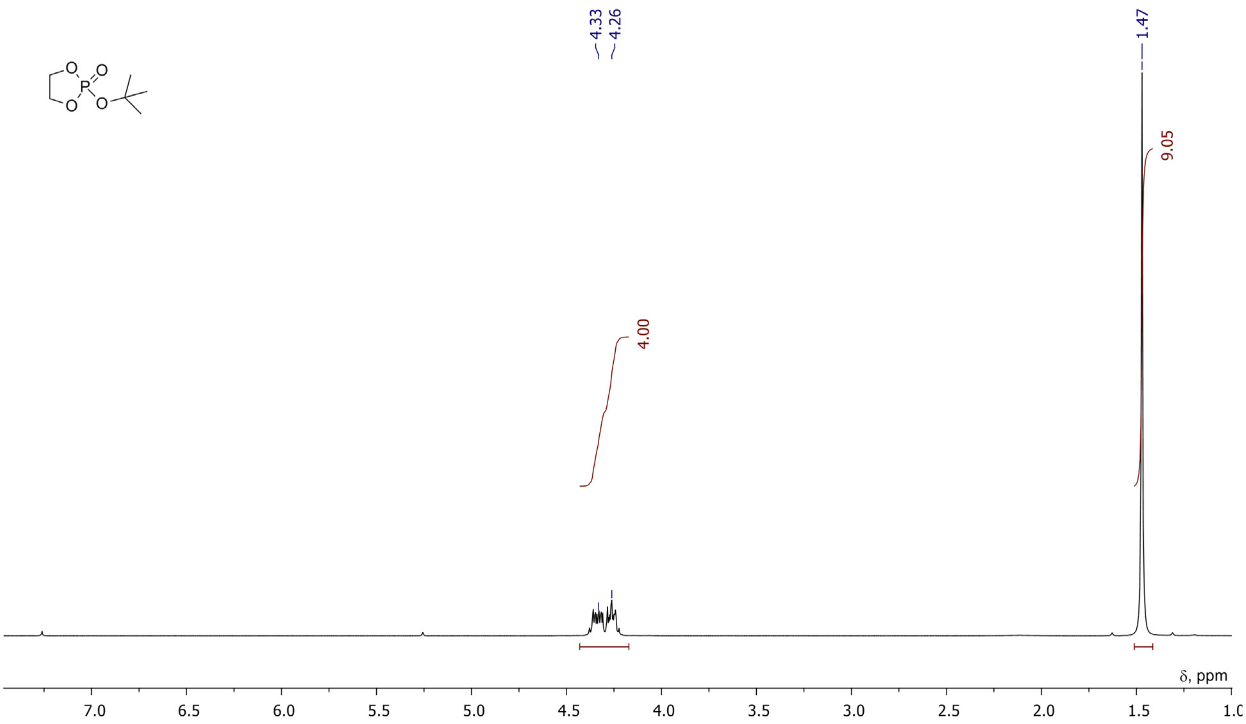

Figure S4. ^{1}H NMR spectrum (CDCl$_3$, 20 °C, 400 MHz) of ^{t}BuOEP.

(*S*)-3-Methyl-1,4-dioxane-2,5-dione (*L*-methylglycolide, *L*-MeGL)

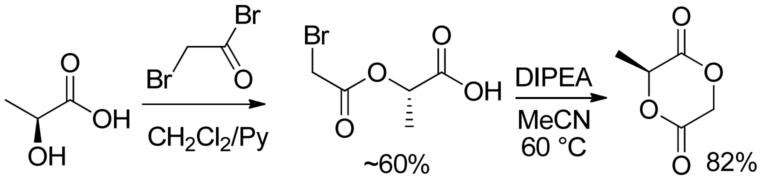

L-MeGL was prepared as described previously [4,5], the yield of polymerization grade product (Figure S2) was 82%.

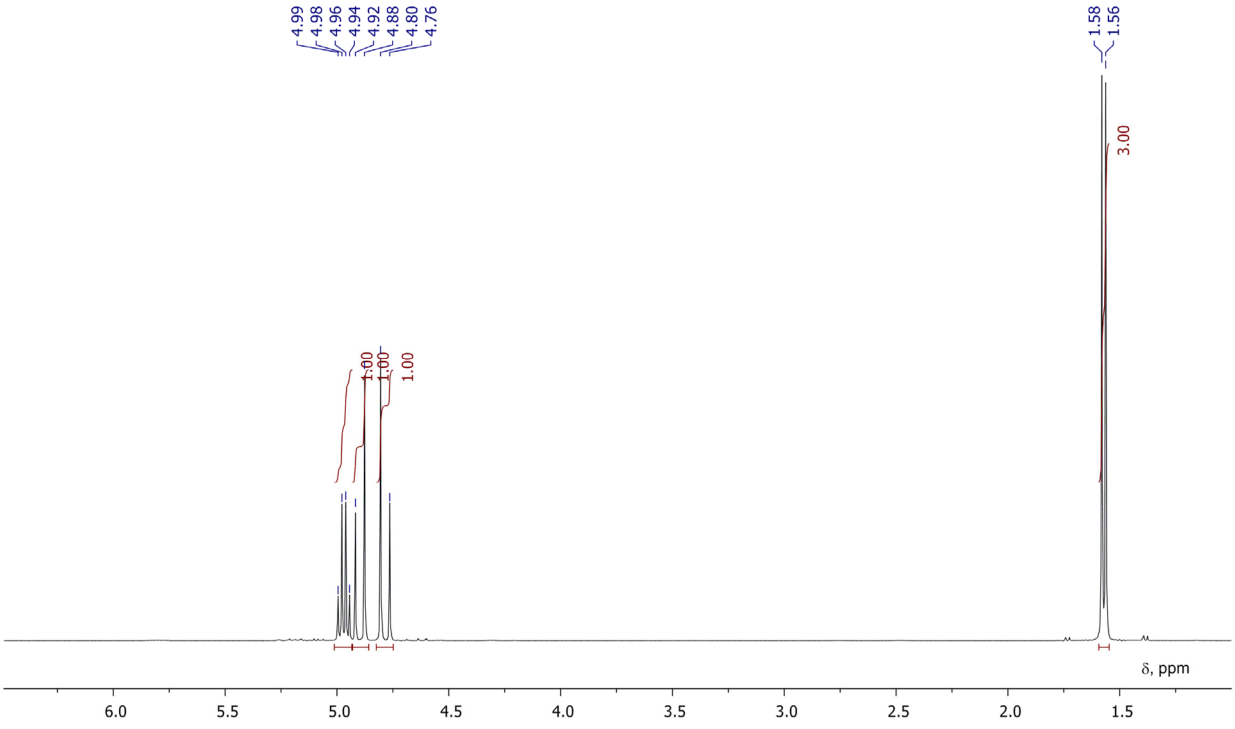

Figure S5. ^{1}H NMR spectrum ($CDCl_3$, 20 °C, 400 MHz) of *L*-MeGL.

S2.2. NMR Spectra of (Co)polymers

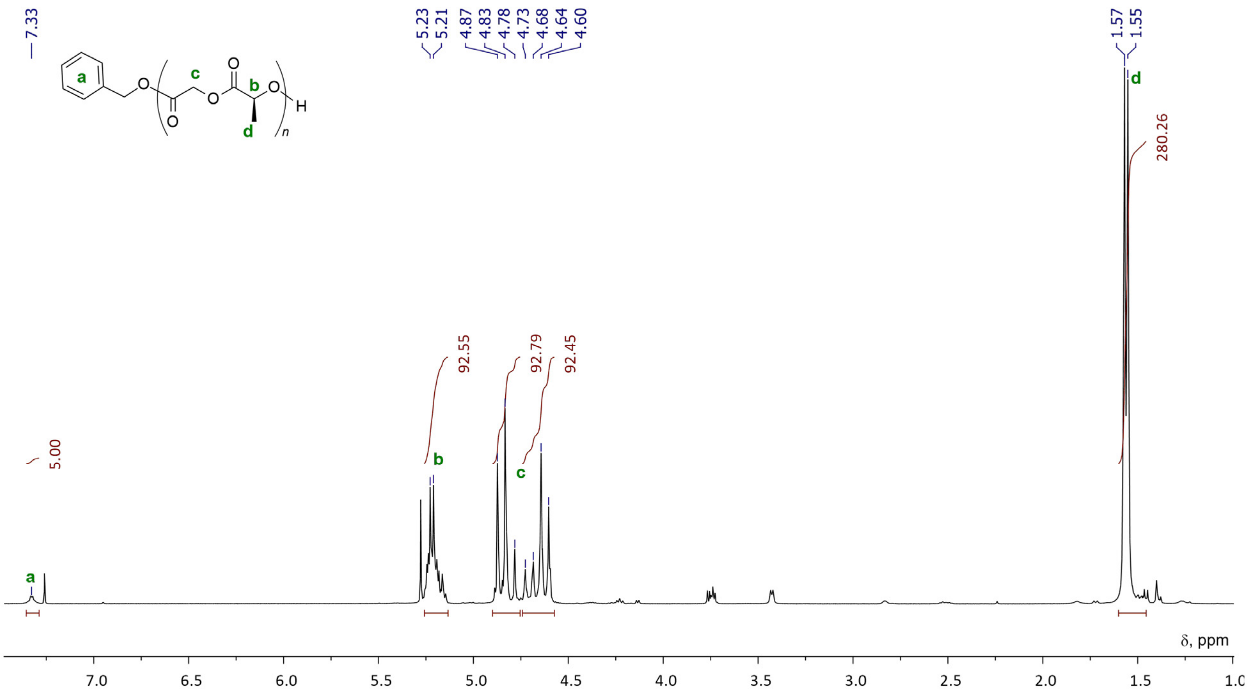

Figure S6. ^{1}H NMR spectrum ($CDCl_3$, 20 °C, 400 MHz) of poly(*L*-MeGL).

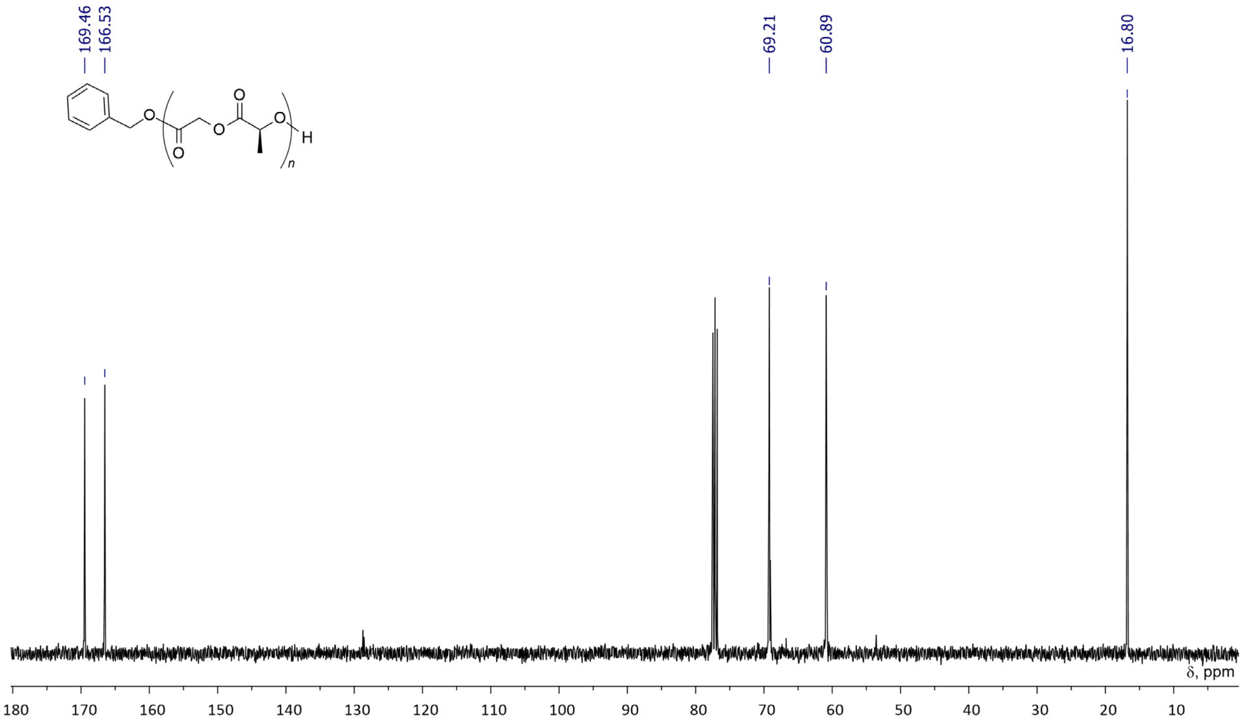

Figure S7. ^{13}C NMR spectrum ($CDCl_3$, 20 °C, 101 MHz) of poly(*L*-MeGL).

^{1}H and ^{31}P NMR spectra of copolymers were used for determination of M_n^{NMR} and comonomer ratios. Attribution of the signals is presented in Figures S5–S16.

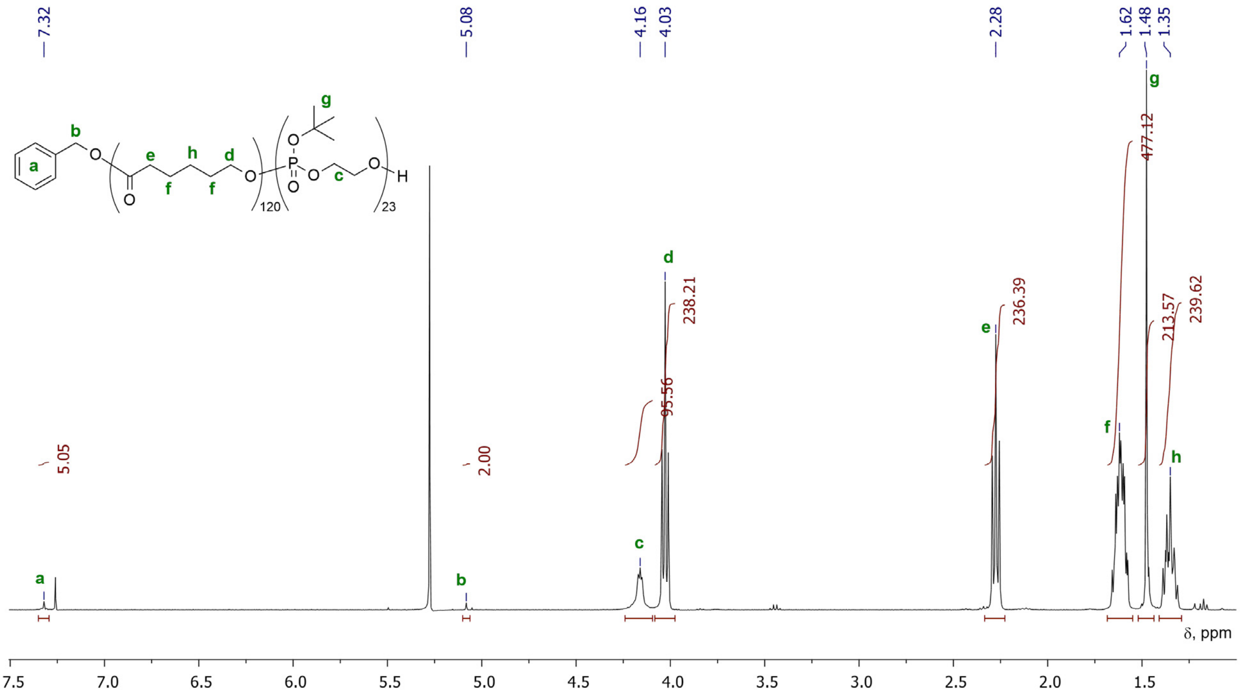

Figure S8. ^{1}H NMR spectrum ($CDCl_3$, 20 °C, 400 MHz) of C1.

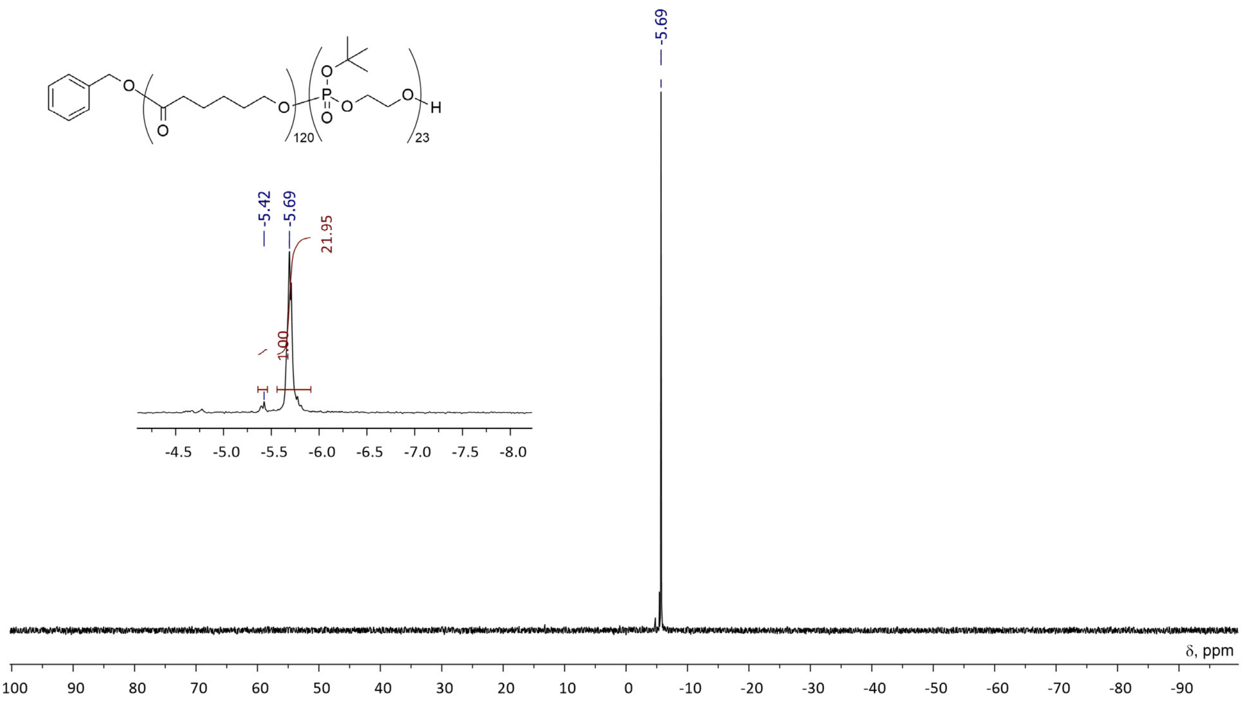

Figure S9. ^{13}P NMR spectrum ($CDCl_3$, 20 °C, 162 MHz) of C1.

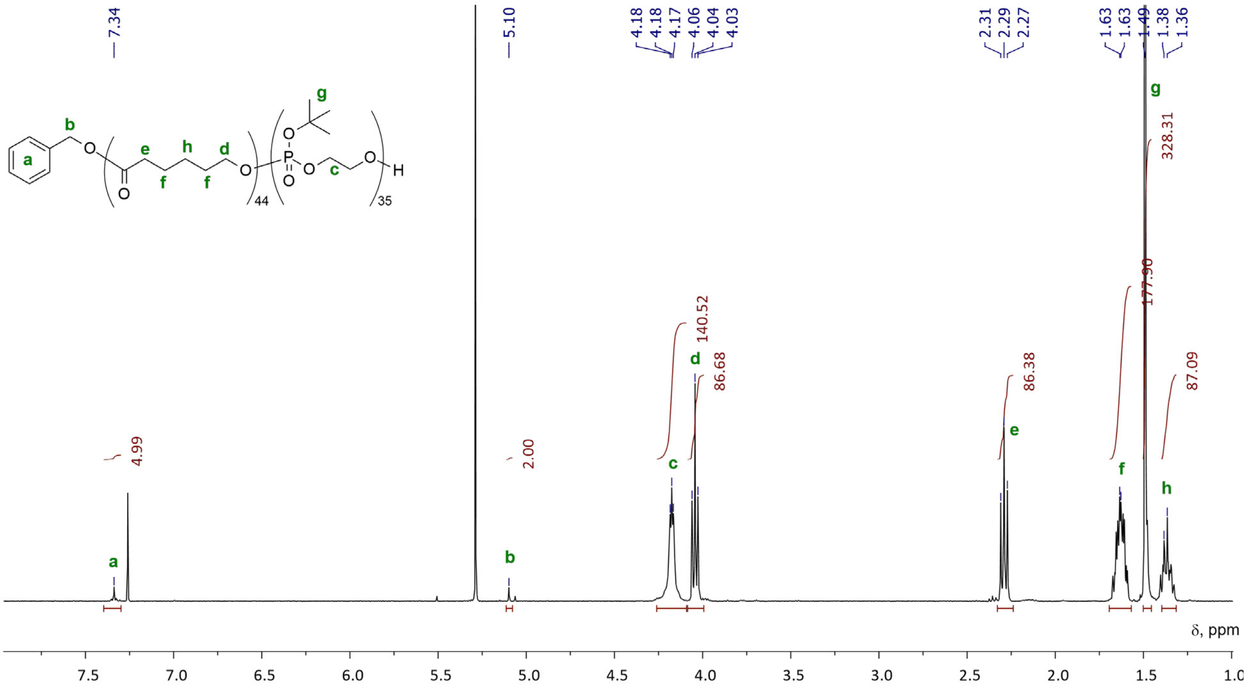

Figure S10. ^{1}H NMR spectrum ($CDCl_3$, 20 °C, 400 MHz) of C2.

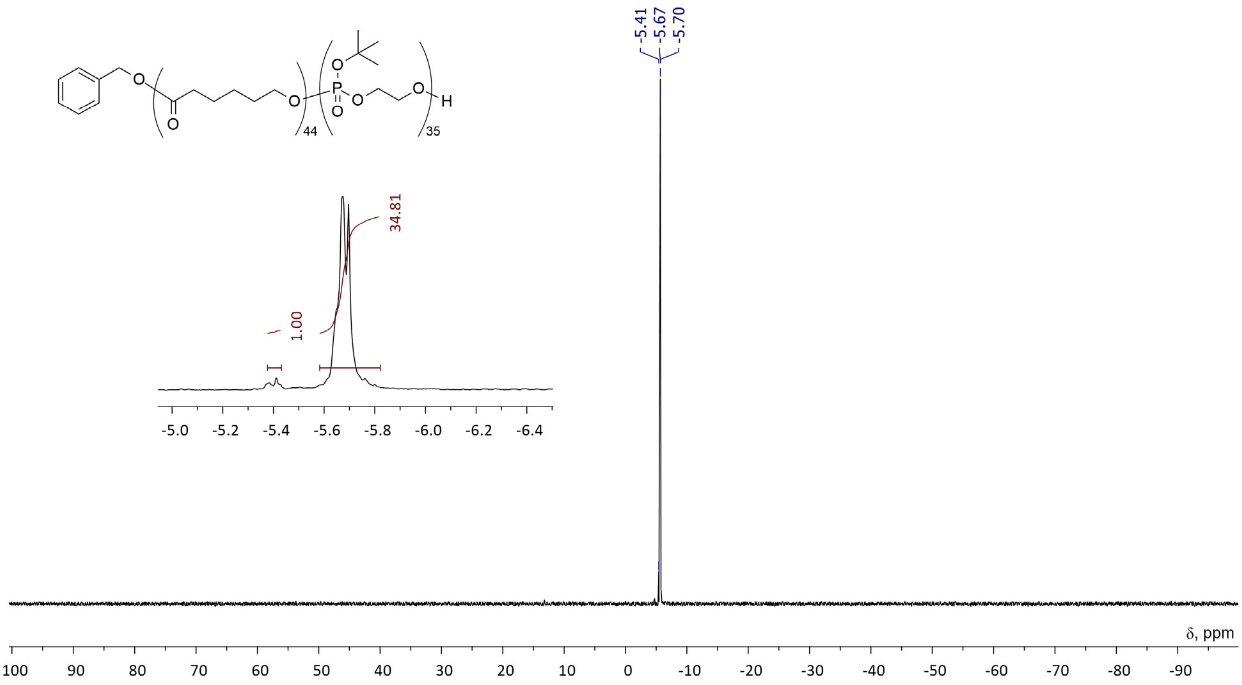

Figure S11. ^{13}P NMR spectrum ($CDCl_3$, 20 °C, 162 MHz) of C2.

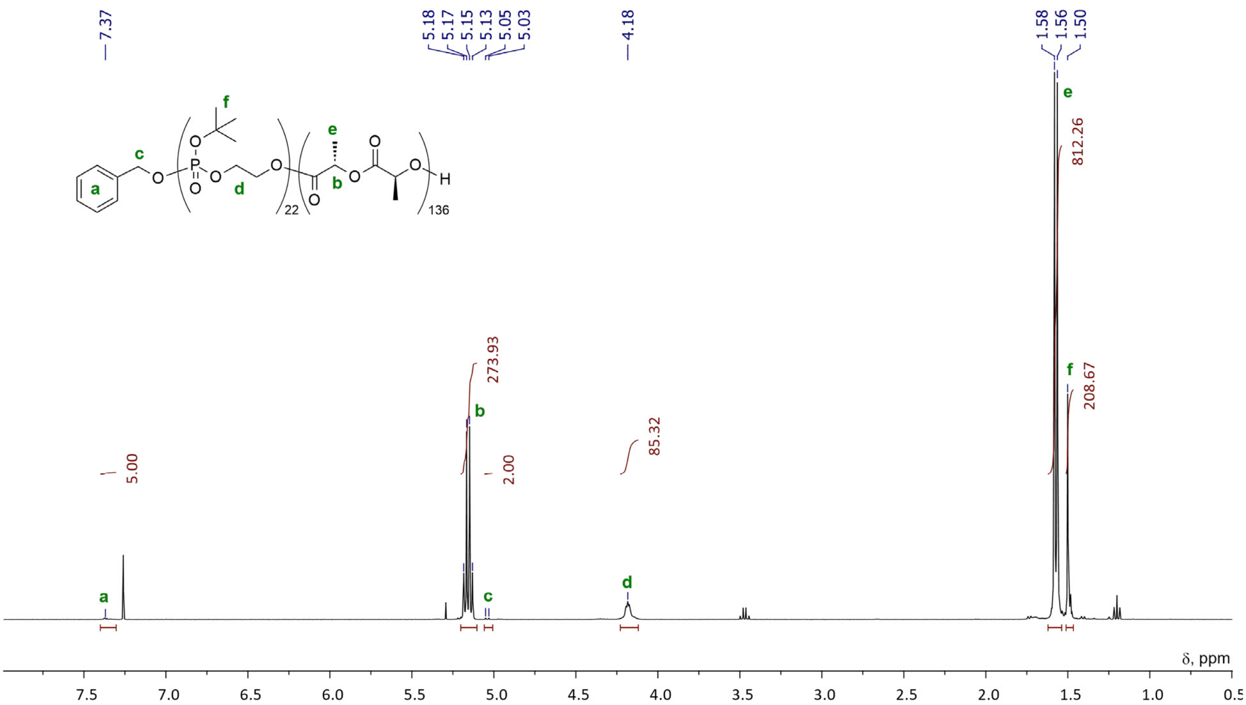

Figure S12. ^{1}H NMR spectrum ($CDCl_3$, 20 °C, 400 MHz) of C3.

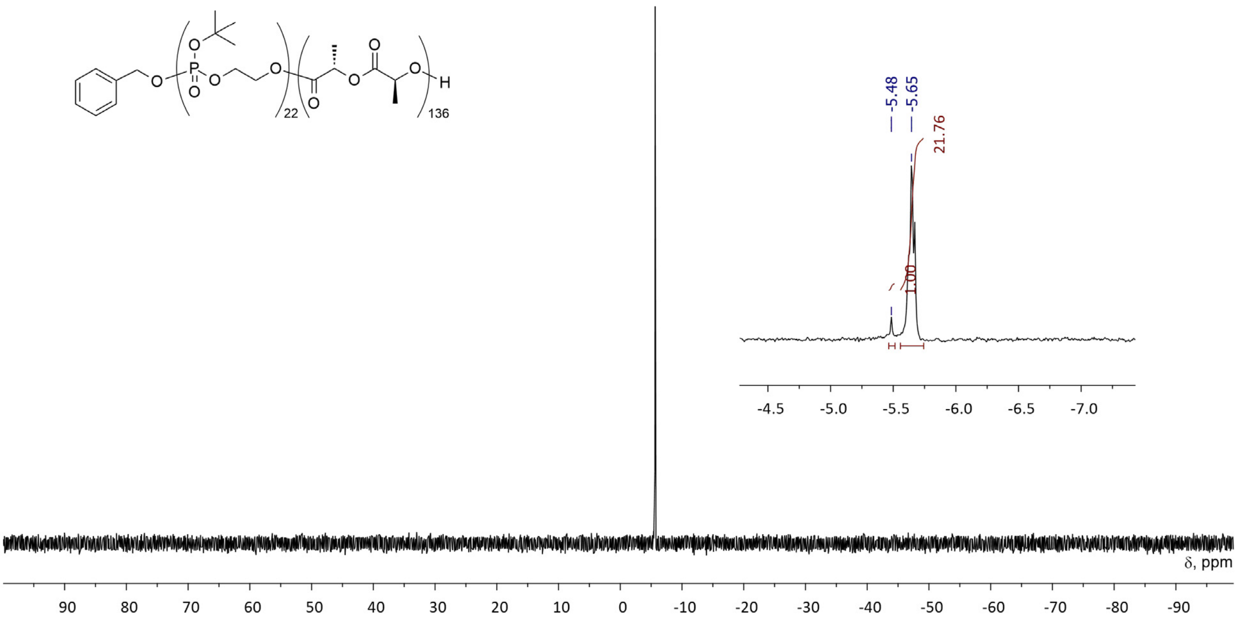

Figure S13. ^{13}P NMR spectrum ($CDCl_3$, 20 °C, 162 MHz) of C3.

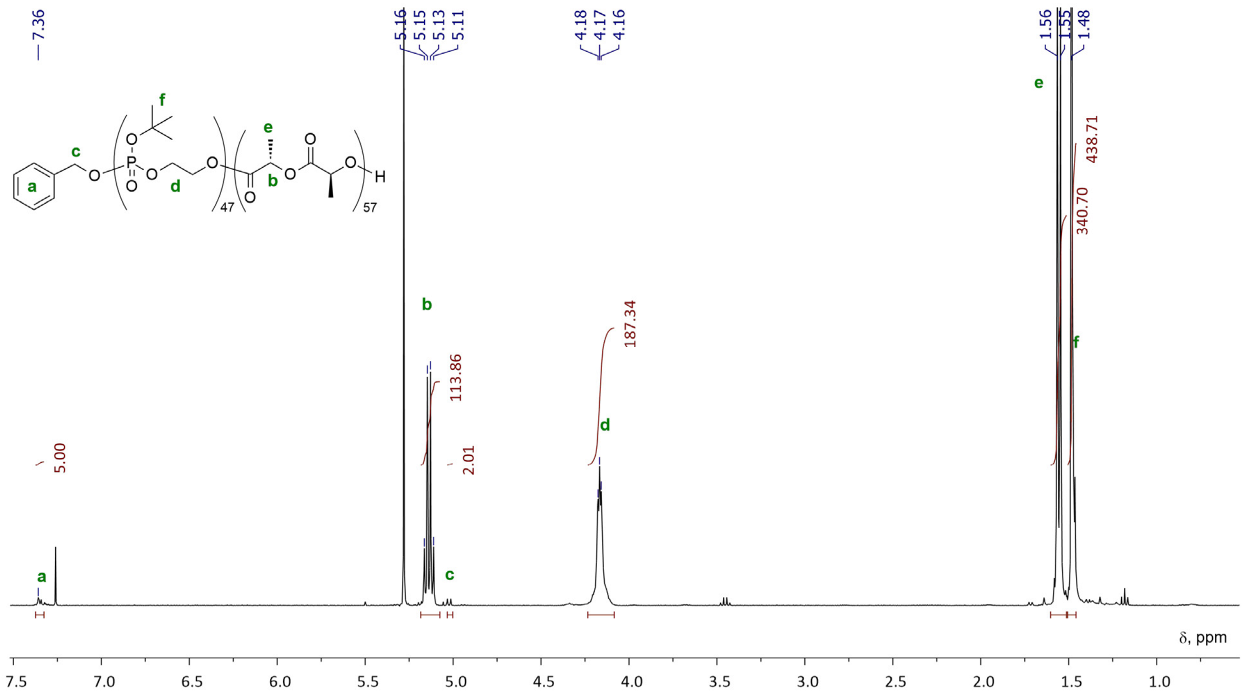

Figure S14. ^{1}H NMR spectrum ($CDCl_3$, 20 °C, 400 MHz) of C4.

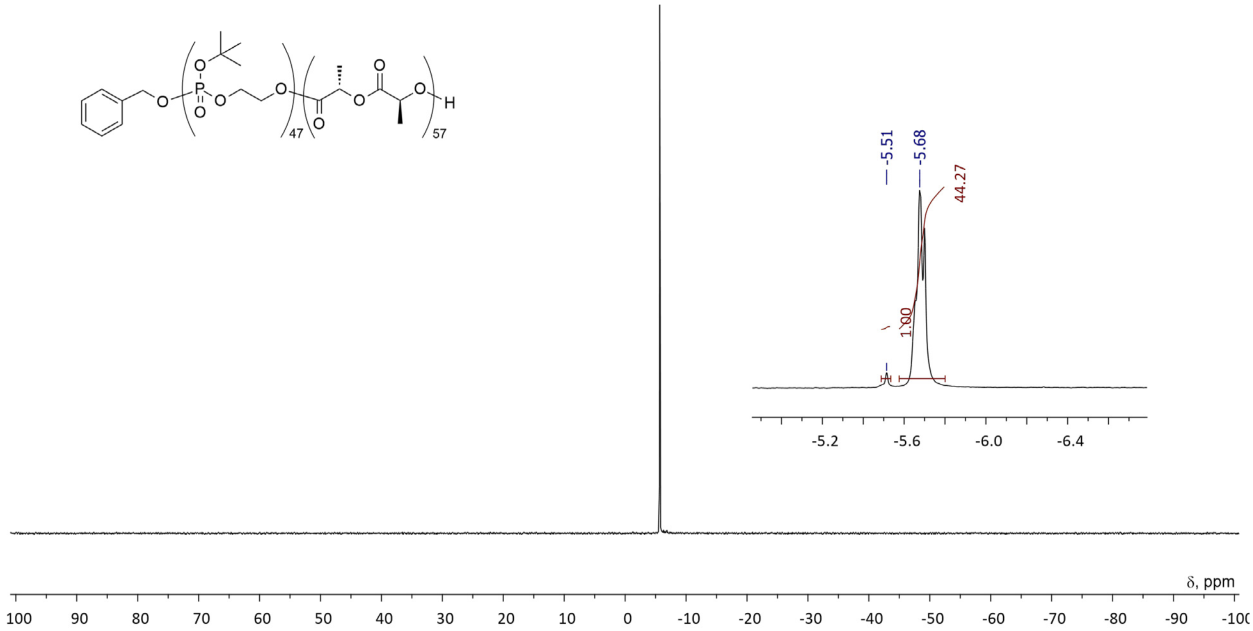

Figure S15 ^{13}P NMR spectrum ($CDCl_3$, 20 °C, 162 MHz) of C4.

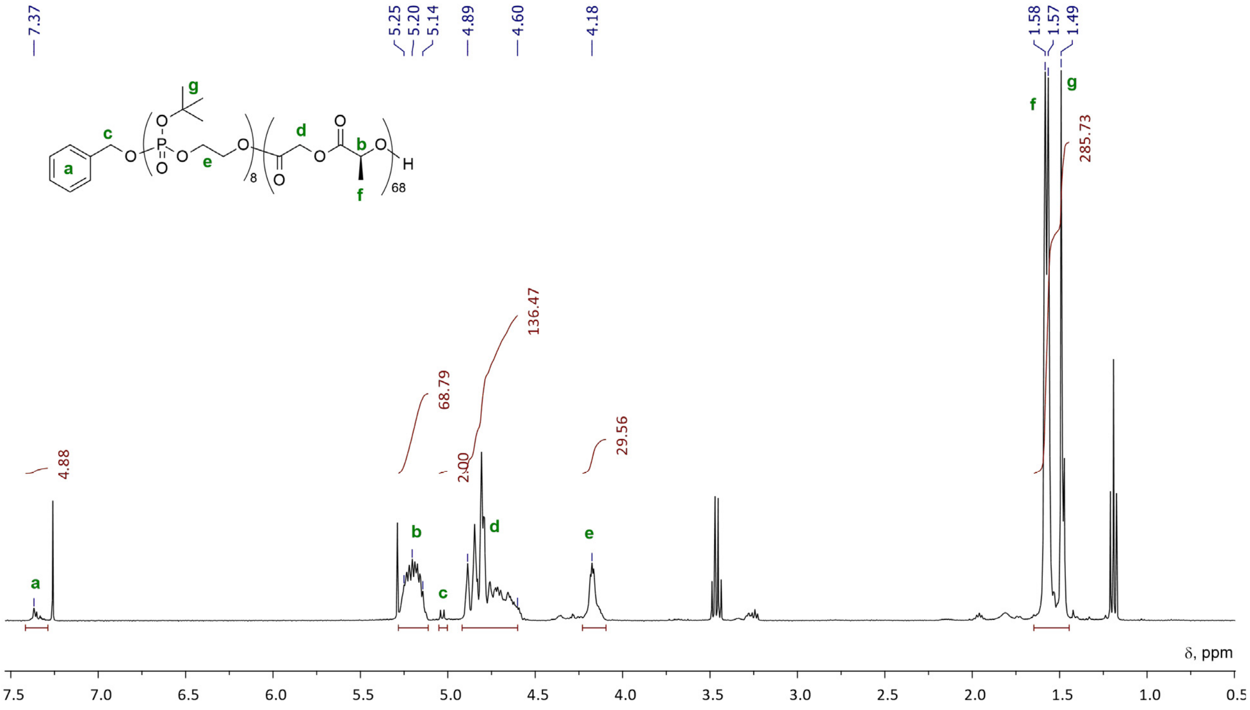

Figure S16. ^{1}H NMR spectrum ($CDCl_3$, 20 °C, 400 MHz) of C5.

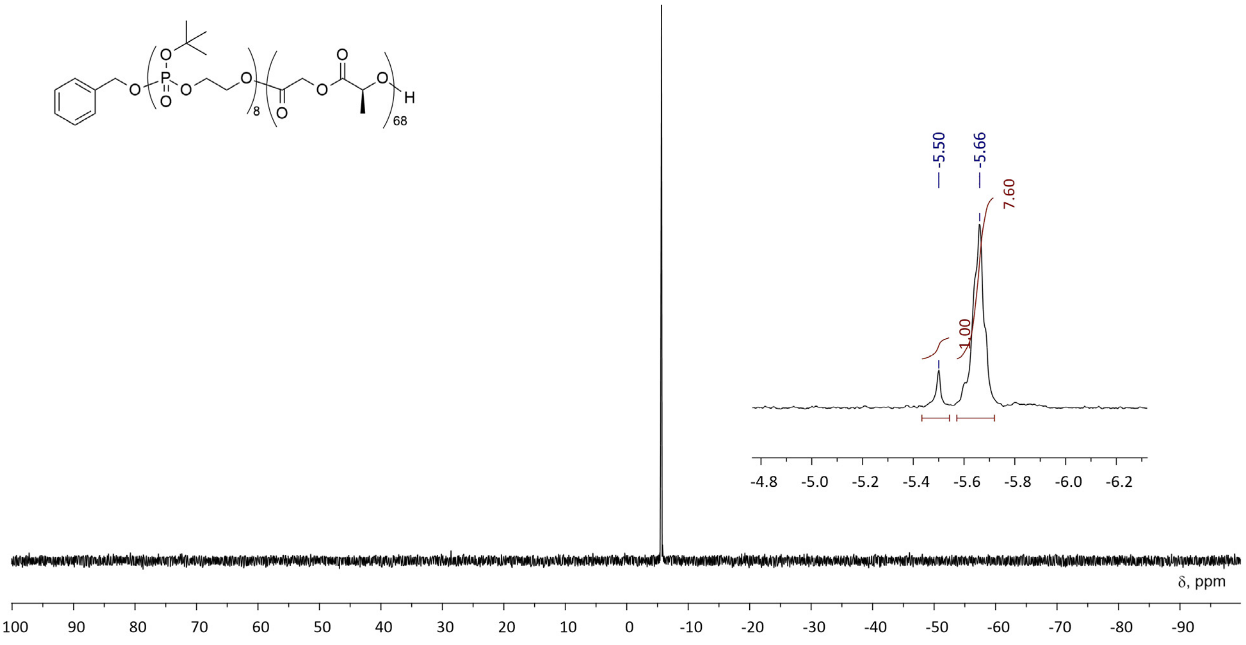

Figure S17^{3}P NMR spectrum ($CDCl_3$, 20 °C, 162 MHz) of C5.

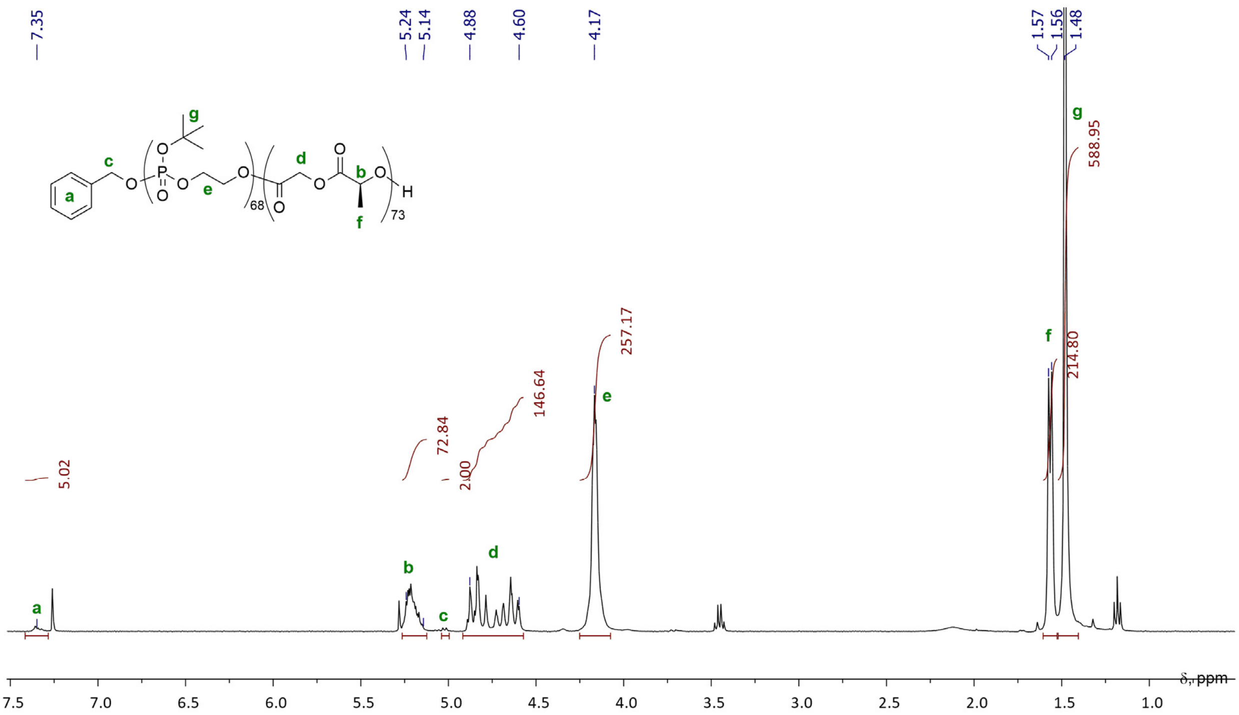

Figure S18. ^{1}H NMR spectrum ($CDCl_3$, 20 °C, 400 MHz) of C6.

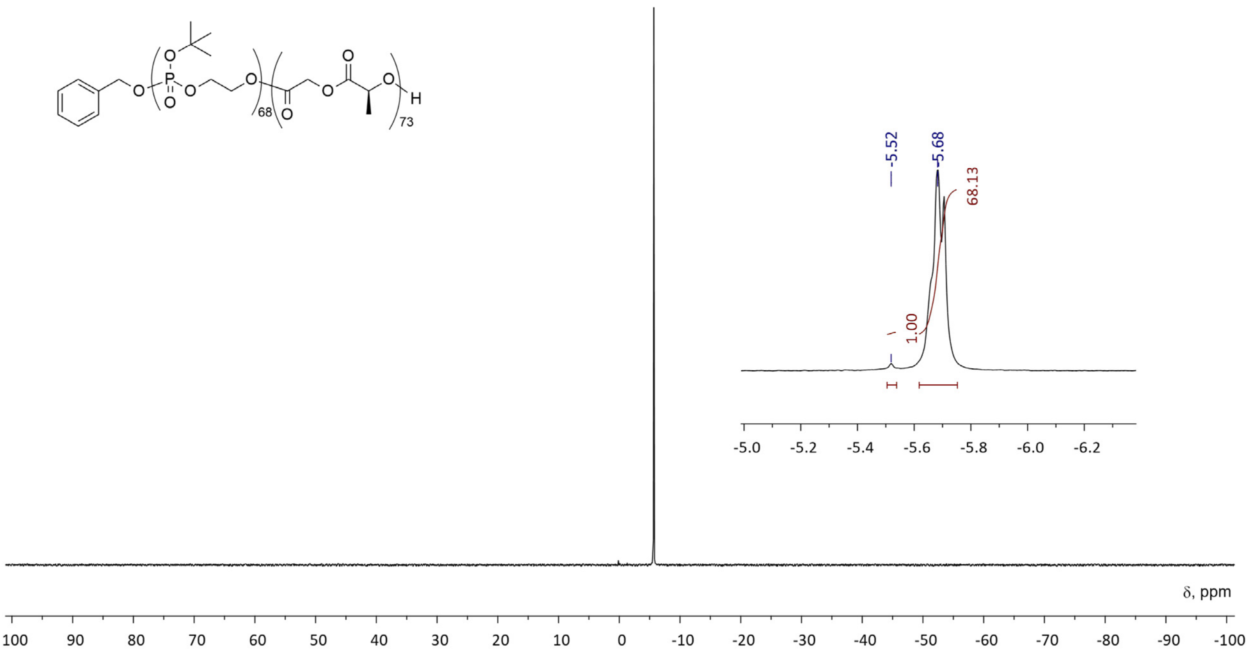

Figure S19. ^{13}P NMR spectrum ($CDCl_3$, 20 °C, 162 MHz) of C6.

S3. Preparation and Laboratory Testing of the Composites

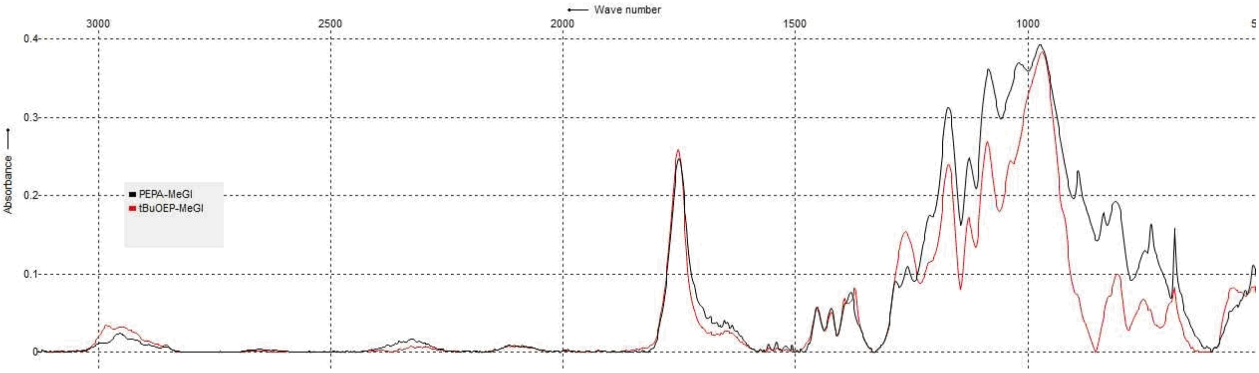

Figure S20. FT-IR spectra of C5 before (red line) and after (black line) heating of 10 wt.% solution in THF (1 h, 80 °C).

S4. Comparative *in vivo* Studies of the Composites

S4.1. First Series of the Experiments

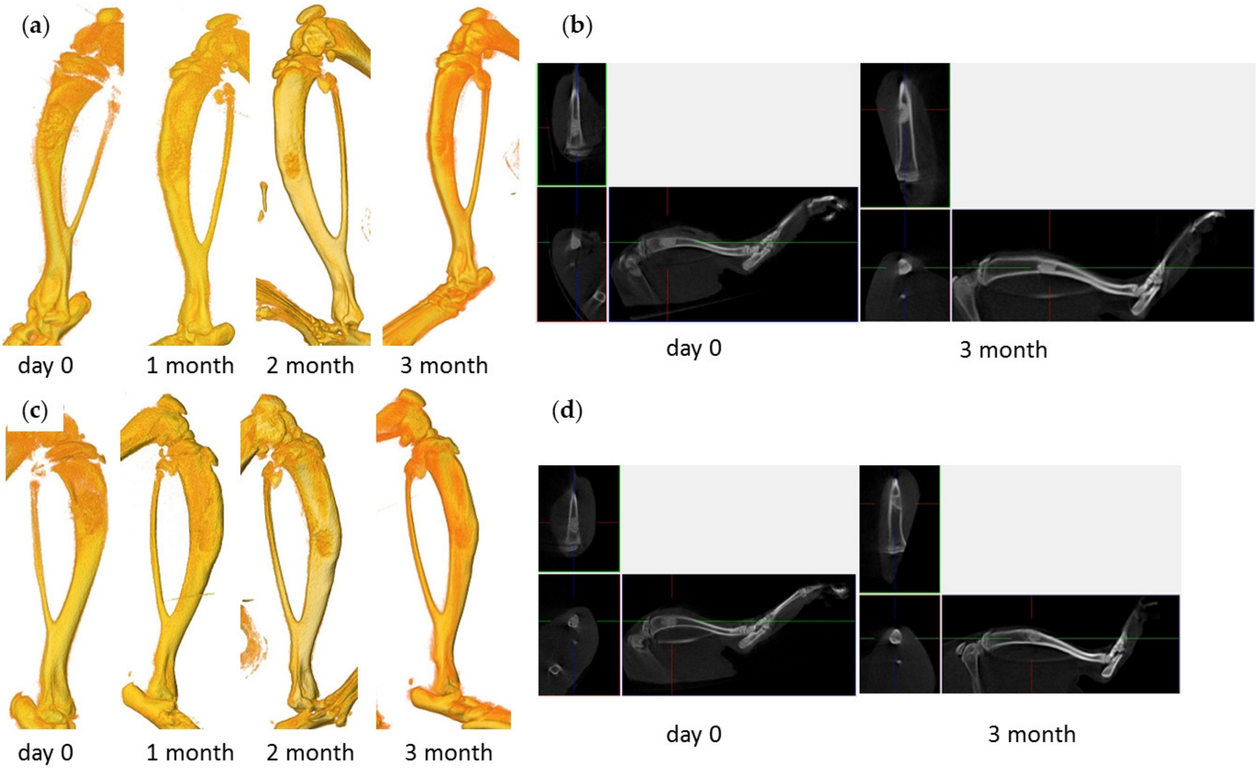

Figure S21. 3D models and orthogonal projections for PCL/pCAp (**a**, **b**) and C2/pCAp (**c**, **d**) implants.

Micro-CT studies do not revealed the marked difference between the results of PCL/pCAp and C2/pCAp implantation.

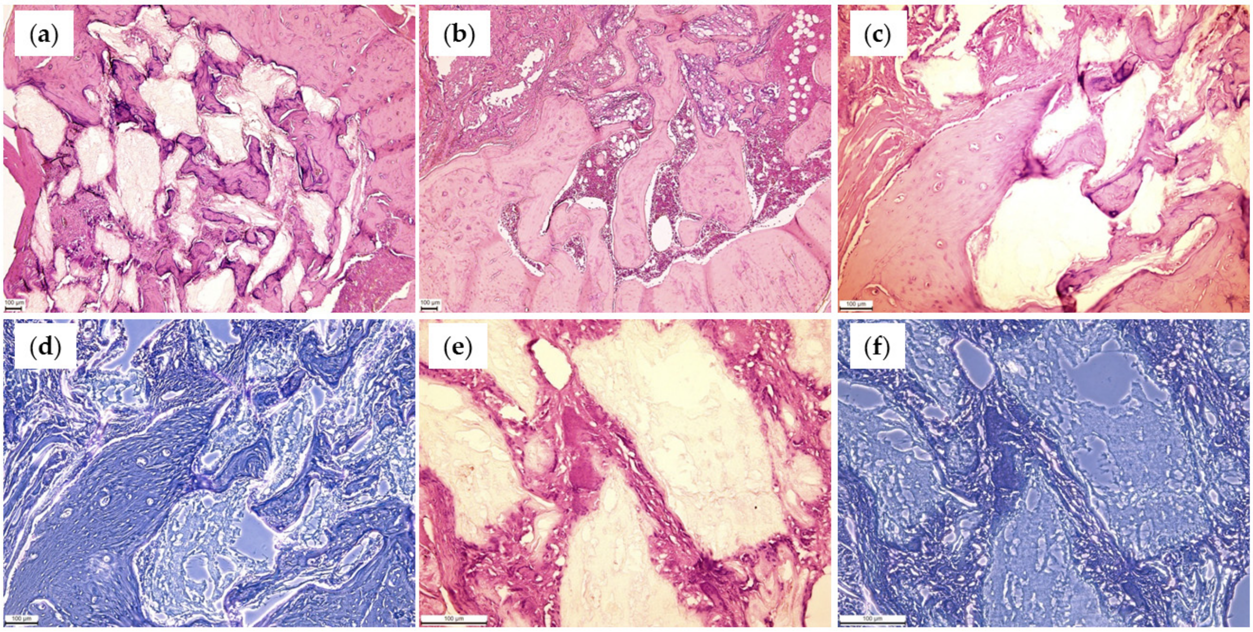

Figure S22. Hematoxylin/eosin stain images of the areas of PCL/pCAp implantation 3 months after the surgery. The images show moderate bone regeneration and weak composite resorption (**a**–**d**); in soft tissues the composite particles were surrounded by MNGCs (**e**, **f**).

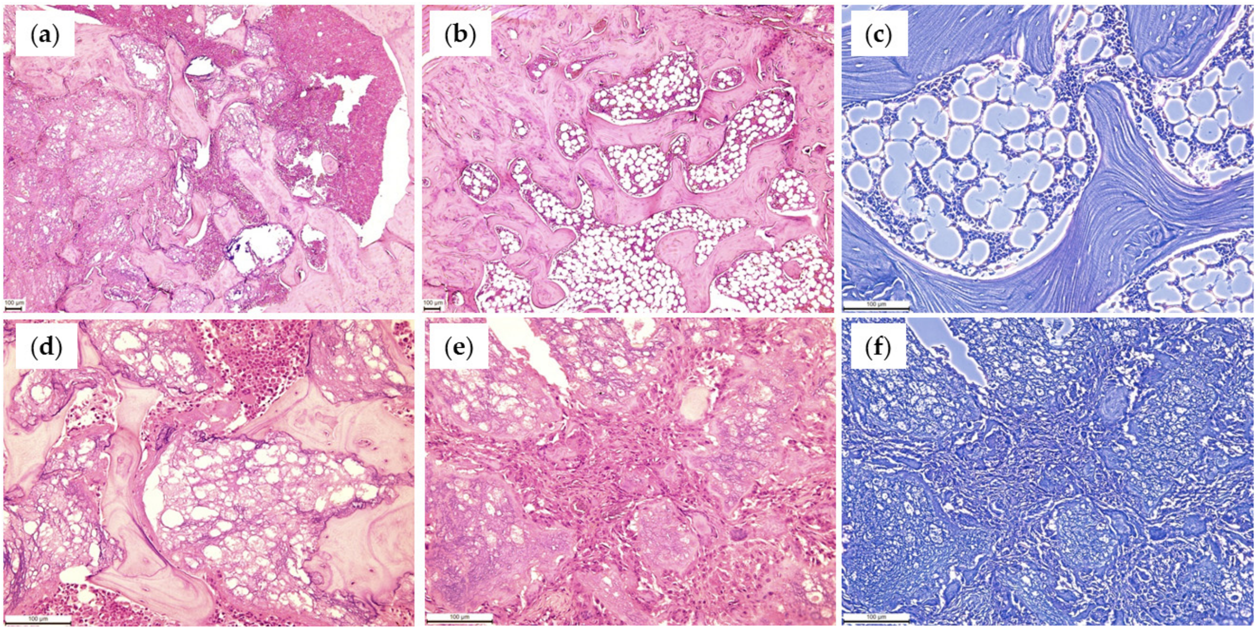

Figure S23. The areas of C2/pCAp implantation 3 months after the surgery. The images show multiple remnants of the composite surrounded by immature trabecular bone (**a**). In part, resorbed composite was replaced by trabecular bone tissue (**b**, **c**); the composite particles were surrounded by MNGCs (**d**) that were observed in surrounding tissues (**e**, **f**).

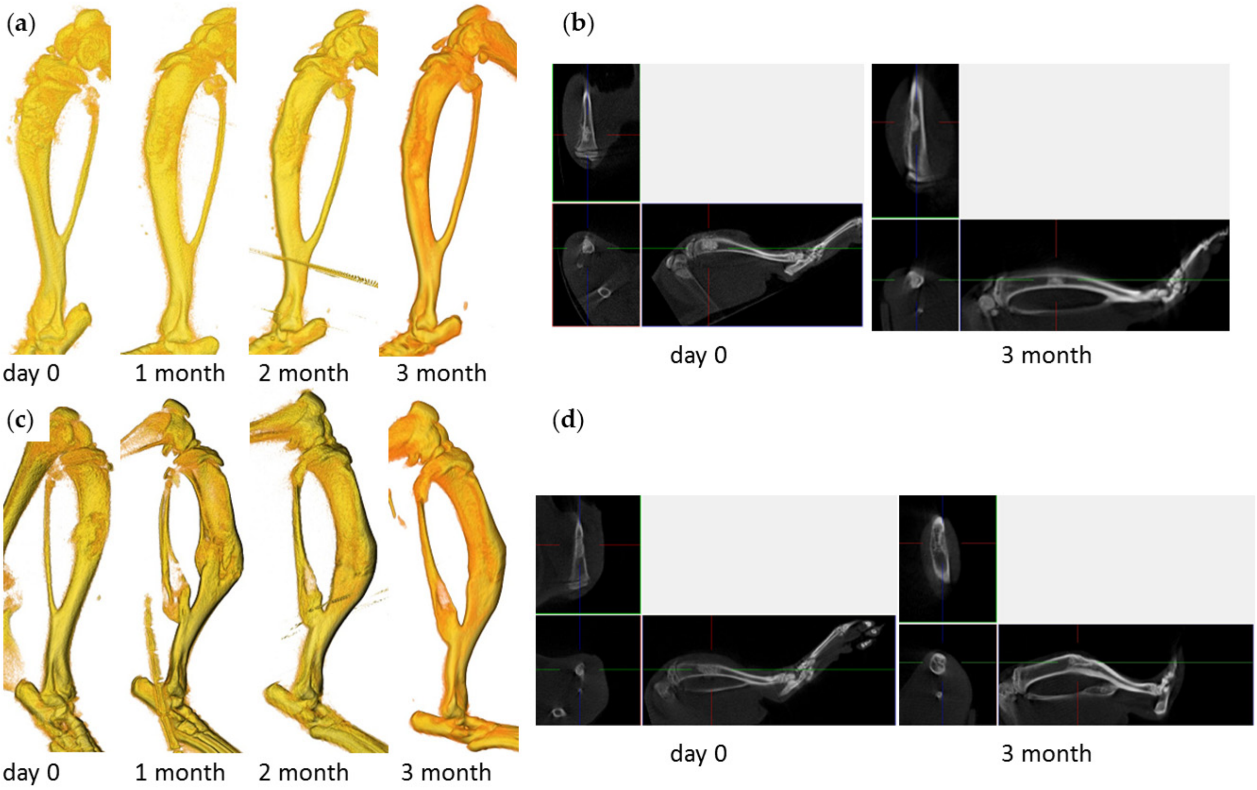

Figure S24. 3D models and orthogonal projections for PLLA/pCAp (**a**, **b**) and C4/pCAp (**c**, **d**) implants.

For PLLA-based composites, micro-CT studies also seemed uninformative.

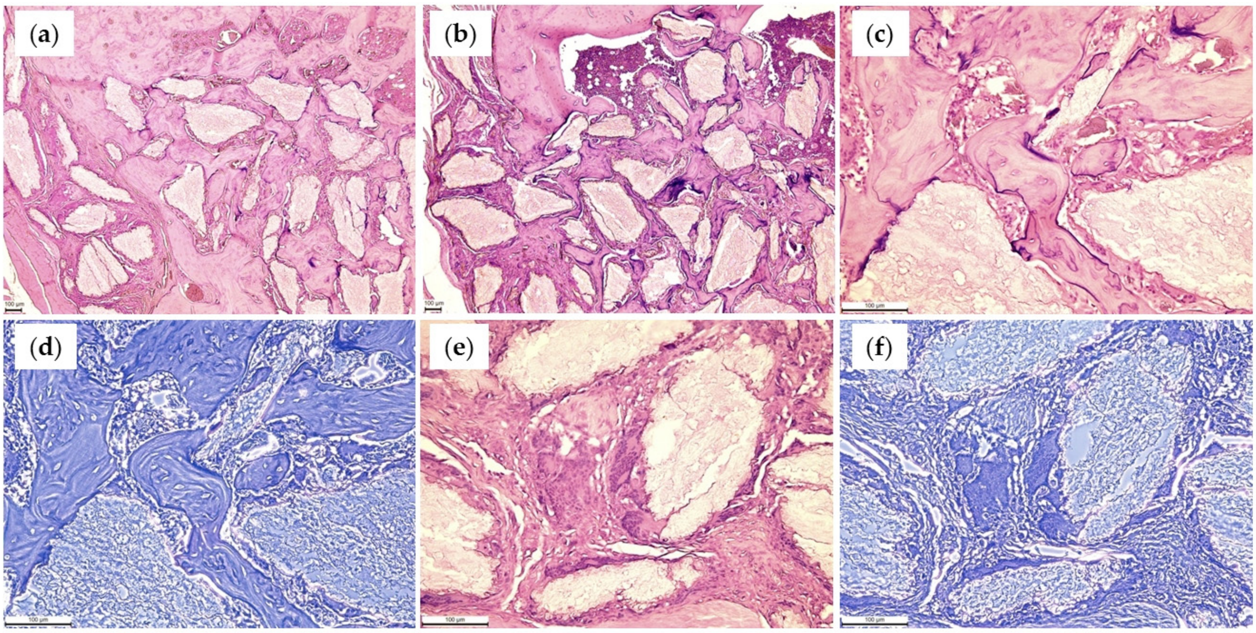

Figure S25. The areas of PLLA/pCAp implantation 3 months after the surgery. The images show good bone regeneration and weak composite resorption (**a**–**d**); in soft tissues the composite particles were surrounded by MNGCs (**e**, **f**).

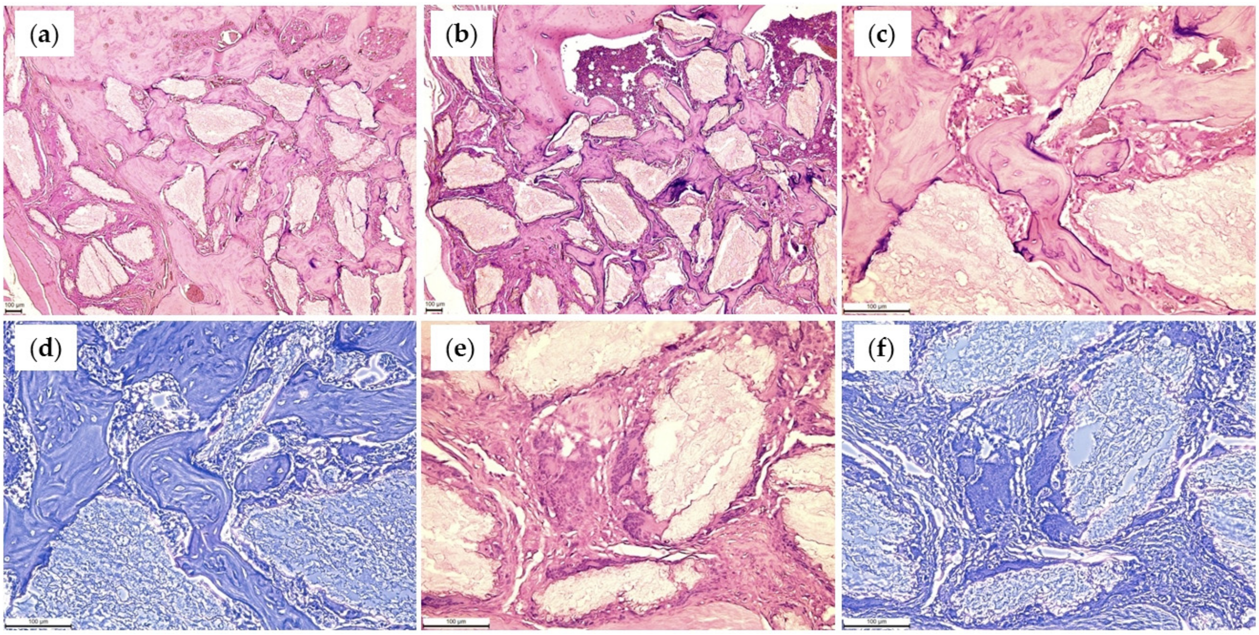

Figure S26. The areas of C3/pCAp implantation 3 months after the surgery. The images show good bone regeneration and weak composite resorption (**a**–**d**); in soft tissues the composite particles were surrounded by MNGCs (**e**, **f**).

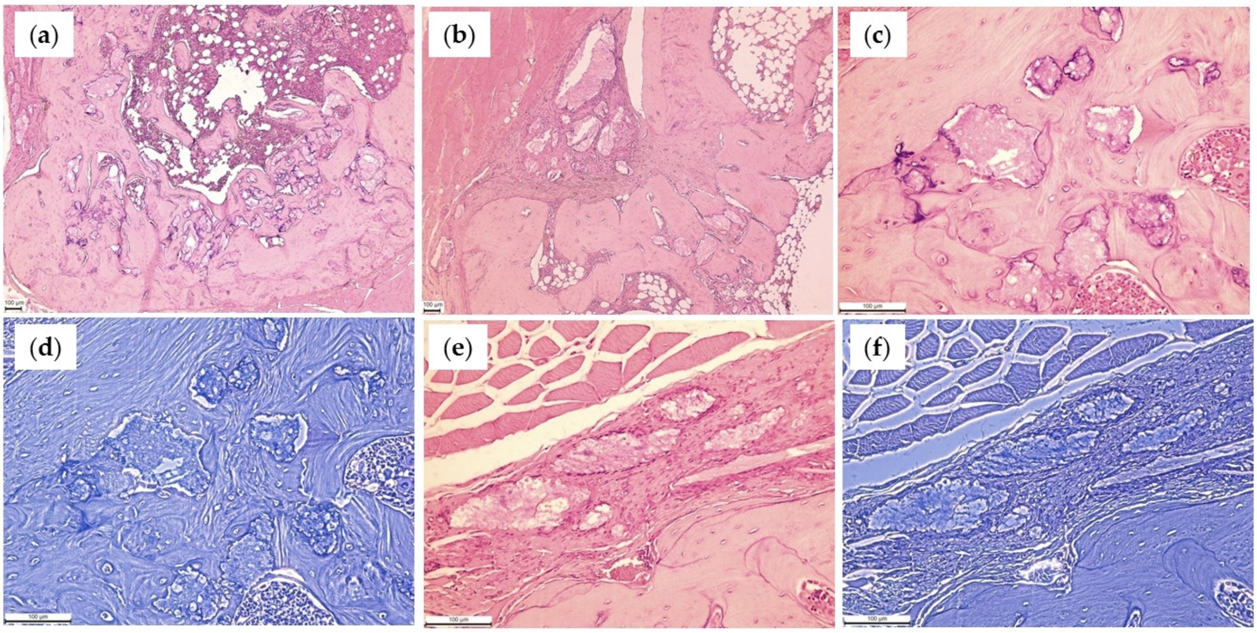

Figure S27. The areas of C4/pCAp implantation 3 months after the surgery. The images show good bone regeneration and composite resorption with a formation of mature bone (**a**–**d**); in soft tissues the composite particles were surrounded by thin macrophagal capsules (**e**, **f**).

S4.2. Second Series of the Experiments

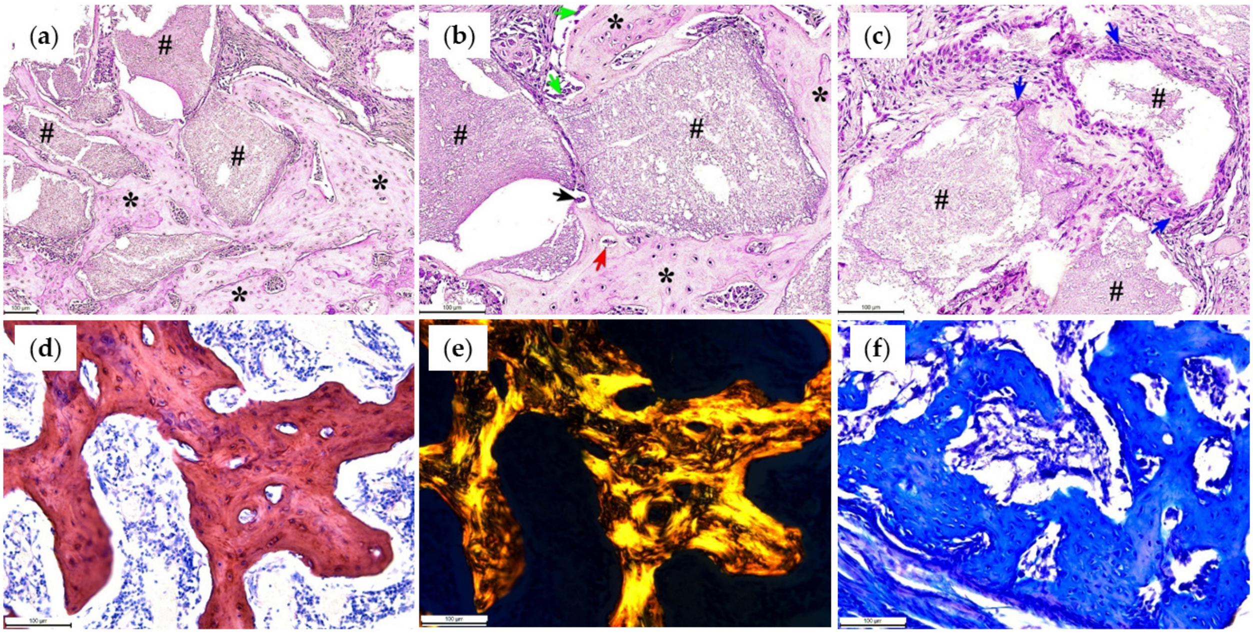

Figure S28. The areas of pCAp implantation 1 months after the surgery. (**a**) The fragments of the implant are surrounded by cancellous immature bone with a large number of osteocytes (light-field microscopy, hematoxylin-eosin staining, magnification 100×). (**b**) The area of bone regeneration surrounding the implant. The beginning of the formation of the Haversian canals is visible (light-field microscopy, hematoxylin-eosin staining, magnification 200×). (**c**) Intensive resorption of implant fragments in soft tissues by macrophages and MNGCs, formation of connective tissue microcapsules around the implant fragments (light-field microscopy, hematoxylin-eosin staining, magnification 200×). (**d**) The trabeculae of the bone regenerate turn red (light-field microscopy, picrosirius red staining, magnification 200×). (**e**) Bright anisotropy of the yellow-red color of immature bone tissue, the forming Haversian canals with the bone plates of osteons concentrically located around them are clearly visible (polarization microscopy, picrosirius red staining, magnification 200×). (**f**) Immature bone tissue is uniformly colored blue (light-field microscopy, Mallory staining, magnification 200×). Symbols: # - implant fragments, * - bone regenerate, the arrows indicate osteoblasts (green), osteoclasts (black), rudiments of Haversian canals (red), MNGCs (blue).

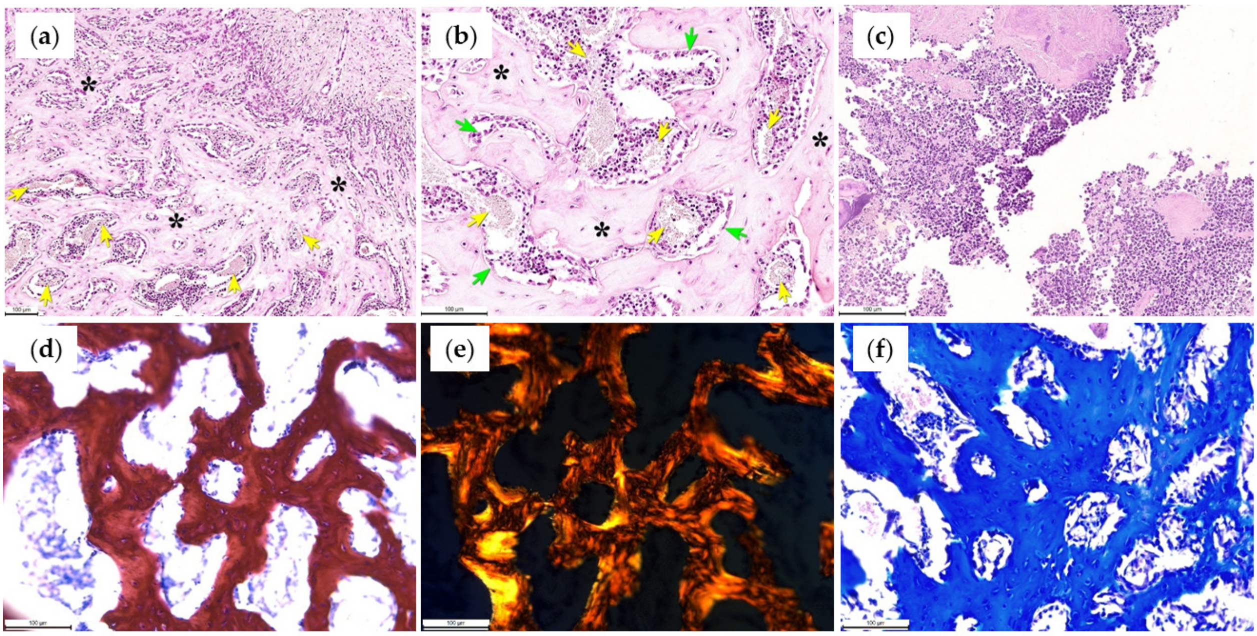

Figure S29. The areas of pCAp-Ce2 implantation 1 month after the surgery. (**a**) Bone regenerate of spongy structure (light-field microscopy, hematoxylin-eosin staining, magnification 100×); (**b**) The area of bone regeneration at a higher magnification, immature bone with a spongy structure with a large number of osteoblasts on the surface of the trabeculae and numerous newly formed blood vessels in the bone marrow cavities (light-field microscopy, hematoxylin-eosin staining, magnification 200×); (**c**) An area with fragments of necrotic bone surrounded by a powerful inflammatory infiltration, mainly neutrophilic (light-field microscopy, hematoxylin-eosin staining, magnification 200×); (**d**) The trabeculae of the bone regenerate are uniformly colored red (light-field microscopy, picrosirius red staining, magnification 200×); (**e**) Bright anisotropy of the yellow-red color of the immature bone, while the forming Haversian channels with the bone plates of the osteon concentrically located around it in the lower left part of the photograph is clearly visible (polarization microscopy, picrosirius red staining, magnification 200×); (**f**) Immature bone is mostly uniformly colored blue (light-field microscopy, Mallory staining, magnification 200×). Symbols: * – bone regenerate, arrows indicate blood vessels (yellow) and osteoblasts (green).

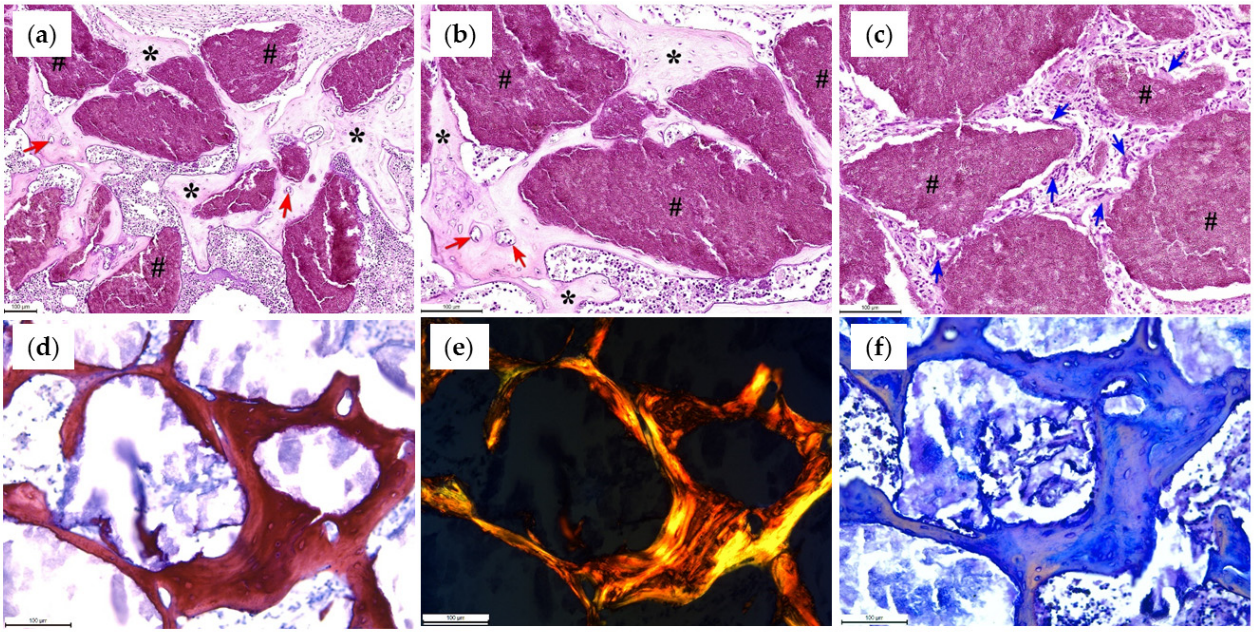

Figure S30. The areas of PLMG/pCAp implantation 1 months after the surgery. (**a**) More intensely colored implant fragments are surrounded by newly formed trabecular bone with a large number of osteocytes (light-field microscopy, hematoxylin-eosin staining, magnification 100×); (**b**) The area of bone regeneration surrounding the implant, at a higher magnification. The beginning of the formation of the Haversian canals is visible (light-field microscopy, hematoxylin-eosin staining, magnification 200×); (**c**) Intensive resorption of implant fragments in soft tissues mainly by MNGCs. Formation of connective tissue microcapsules around the implant fragments (light-field microscopy, hematoxylin-eosin staining, magnification 200×); (**d**) The trabeculae of the bone regenerate turn red (light-field microscopy, picrosirius red staining, magnification 200×); (**e**) Bright anisotropy of the yellow-red color of immature bone tissue, the forming Haversian canals with the bone plates of osteons concentrically located around them are clearly visible (polarization microscopy, picrosirius red staining, magnification 200×); (**f**) Immature bone tissue turns unevenly blue, orange foci are visible (light-field microscopy, Mallory staining, magnification 200×). Designations: # - implant fragments, * - bone regenerate, the arrows indicate the beginnings of Haversian canals (red) and MNGCs (blue).

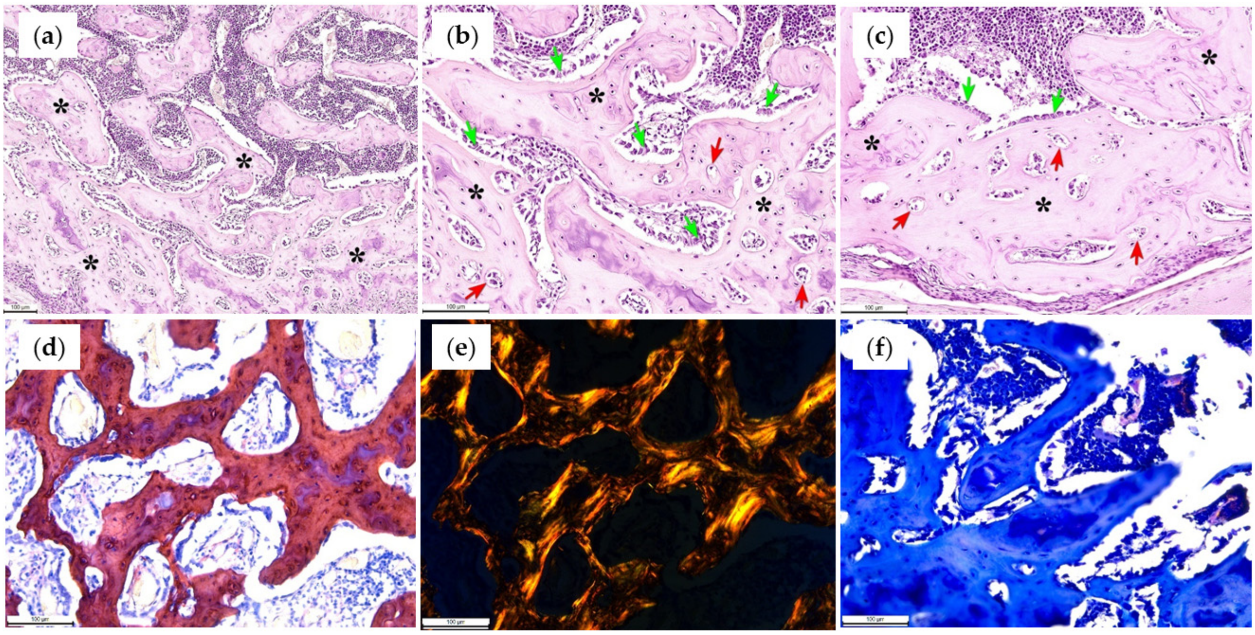

Figure S31. The areas of C6/pCAp implantation 1 months after the surgery. (**a**) Bone regenerate was spongy with a signs of lamellar structure (light-field microscopy, hematoxylin-eosin staining, magnification 100×); (**b**) The area of bone regeneration at a higher magnification. Immature bone with a spongy structure with a large number of osteoblasts on the surface of the trabeculae and rudiments of the Haversian canals (light-field microscopy, hematoxylin-eosin staining, magnification 200×); (**c**) The compaction of the bone matrix increases and the immature bone begins to acquire a lamellar structure with a Haversian system (light-field microscopy, hematoxylin-eosin staining, magnification 200×); (**d**) The trabeculae of the bone regenerate are colored unevenly in red, and remnants of the mineralized cartilage matrix are visible (light-field microscopy, picrosirius red staining, magnification 200×); (**e**) Less vivid anisotropy of the yellow-red color of the immature bone, which may be due to the greater maturity of its bone matrix (polarization microscopy, picrosirius red staining, magnification 200×); (**f**) Immature bone is mostly uniformly colored blue, orange foci are rare, and rudiments of Haversian canals are visible (light-field microscopy, Mallory staining, magnification 200×). Symbols: * - bone regenerate, arrows indicate the rudiments of the Haversian canals (red) and osteoblasts (green).

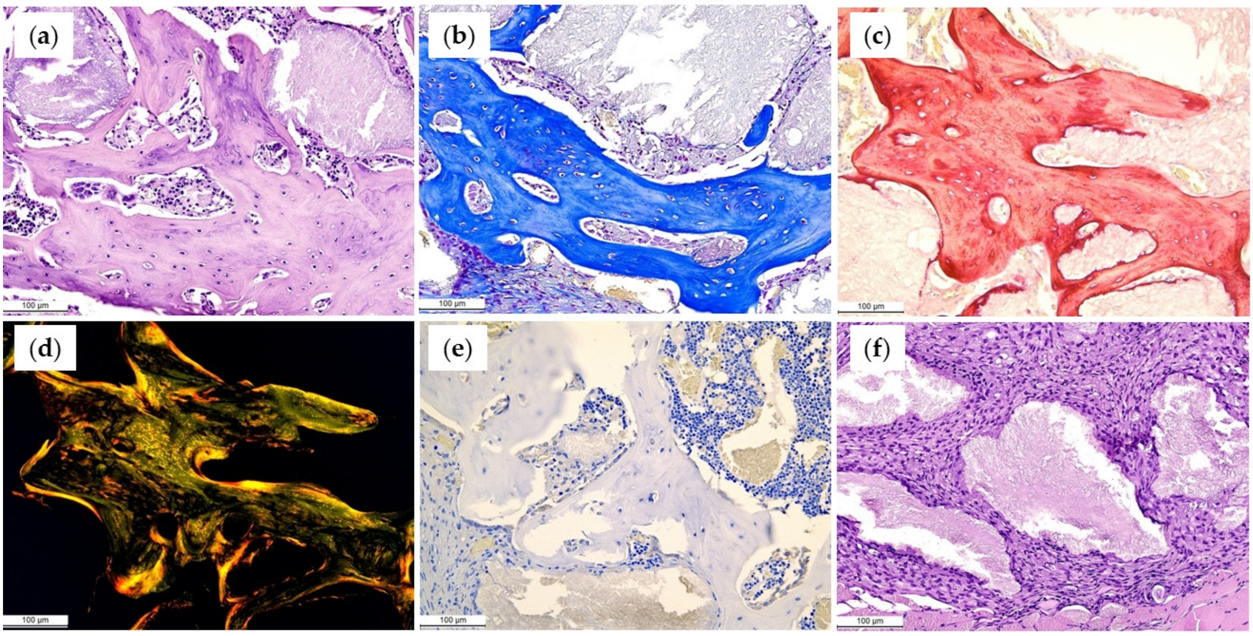

Figure S32. The areas of PLMG/pCAp-Ce1 implantation 1 month after the surgery. (**a**) The fragments of the implant of a fine-fibrous structure are surrounded by spongy bone tissue with a signs of lamellar structure (light-field microscopy, hematoxylin-eosin staining, magnification 200×); (**b**) Immature bone is uniformly colored blue (light-field microscopy, Mallory staining, magnification 200×); (**c**) The trabeculae of the bone regenerate turn red (light-field microscopy, picrosirius red staining, magnification 200×); (**d**) Bright anisotropy of the yellow-red color of immature bone tissue, the ordered arrangement of collagen fibers and the forming Haversian canals are visible (polarization microscopy, picrosirius red staining, magnification 200×); (**e**) Expression of alkaline phosphatase in osteoblasts (colored brown) on the surface of the trabeculae of bone regenerate. Light-field microscopy, immunohistochemical staining for alkaline phosphatase, magnification 200×); (**f**) Formation of connective tissue microcapsules around the implant fragments (light-field microscopy, hematoxylin-eosin staining, magnification 200×).

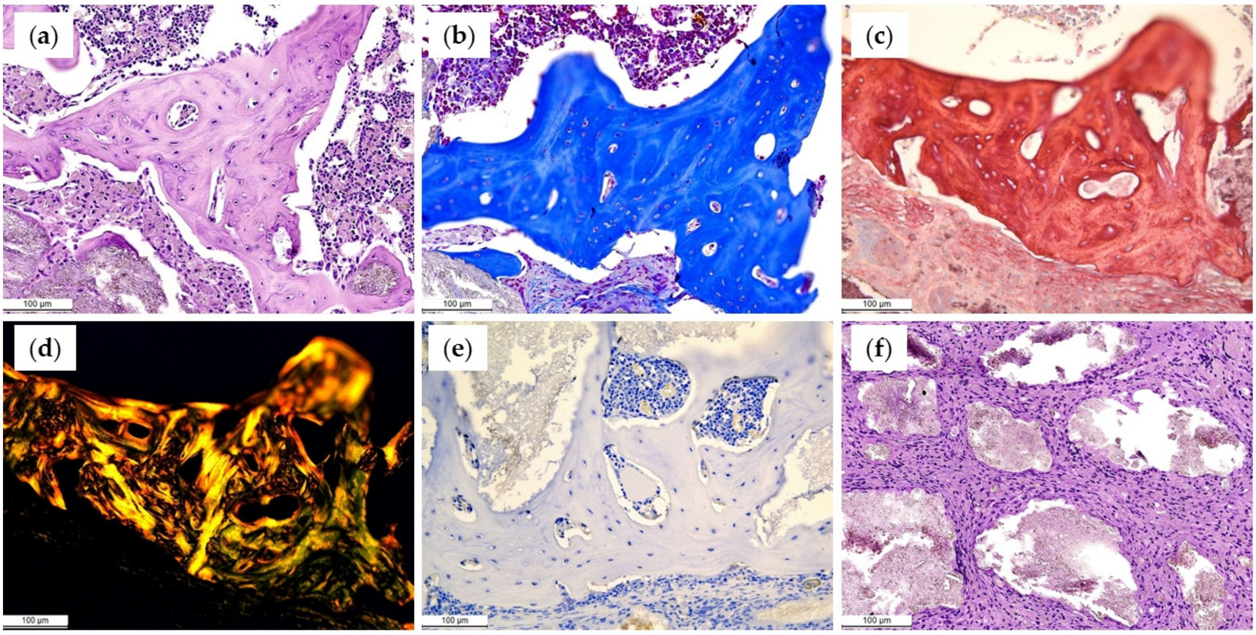

Figure S33. The areas of PLMG/pCAp-Ce2 implantation 1 month after the surgery. (**a**) Fragments of the implant of a thin-fibrous structure are surrounded by lamellar tissue with a signs of compact structure (light-field microscopy, hematoxylin-eosin staining, magnification 200×); (**b**) Immature bone is uniformly colored blue (light-field microscopy, Mallory staining, magnification 200×); (**c**) The trabeculae of the bone regenerate turn red (light-field microscopy, picrosirius red staining, magnification 200×); (**d**) Bright anisotropy of the yellow-red color of immature bone tissue, the ordered arrangement of collagen fibers and the formed Haversian canals are clearly visible (polarization microscopy, picrosirius red staining, magnification 200×); (**e**) Expression of alkaline phosphatase in osteoblasts (colored brown) on the surface of the trabeculae of bone regenerate (light-field microscopy, immunohistochemical staining for alkaline phosphatase, magnification 200×); (**f**) Formation of connective tissue microcapsules around the implant fragments (light-field microscopy, hematoxylin-eosin staining, magnification 200×).

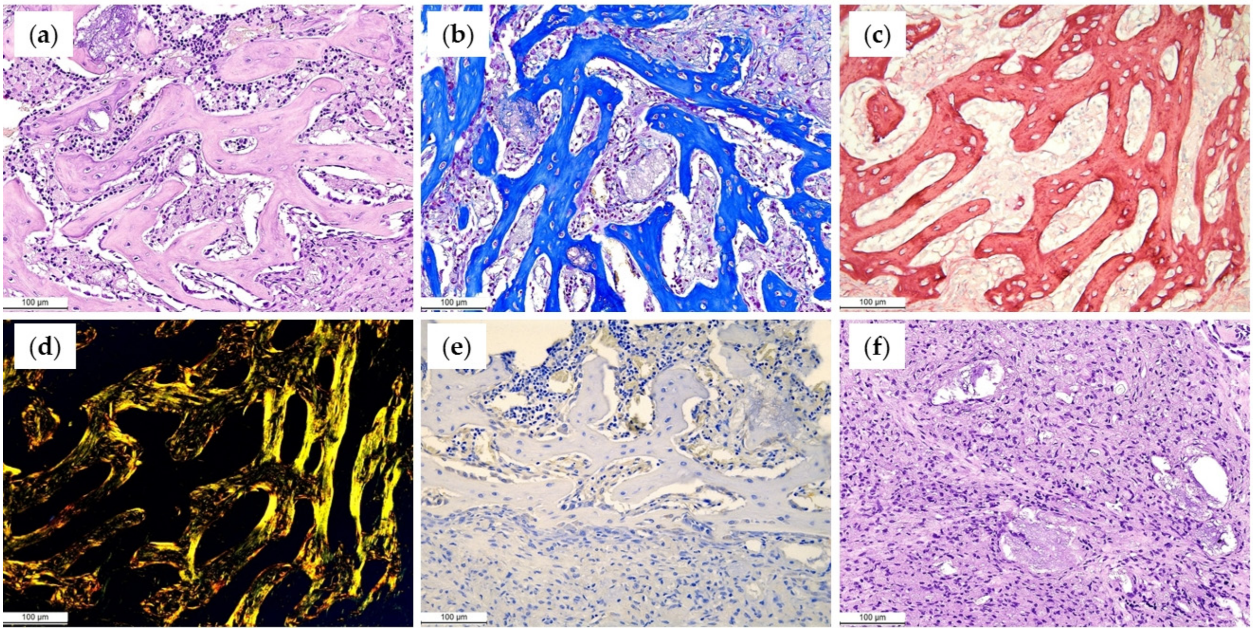

Figure S34. The areas of C6/pCAp-Ce1 implantation 1 month after the surgery. (**a**) The fragments of the implant have a fine fibrous structure surrounded by immature spongy bone tissue (light-field microscopy, hematoxylin-eosin staining, magnification 200×); (**b**) Immature bone is uniformly colored blue (light-field microscopy, Mallory staining, magnification 200×); (c) The trabeculae of the bone regenerate turn red (light-field microscopy, picrosirius red staining, magnification 200×); (**d**) Bright anisotropy of the yellow-red color of immature bone tissue, the ordered arrangement of collagen fibers is clearly visible (polarization microscopy, picrosirius red staining, magnification 200×); (**e**) Expression of alkaline phosphatase in osteoblasts (colored brown) on the surface of the trabeculae of bone regenerate (light-field microscopy, immunohistochemical staining for alkaline phosphatase, magnification 200×); (**f**) Intensive implant resorption and replacement with fibrous tissue (light-field microscopy, hematoxylin-eosin staining, magnification 200×).

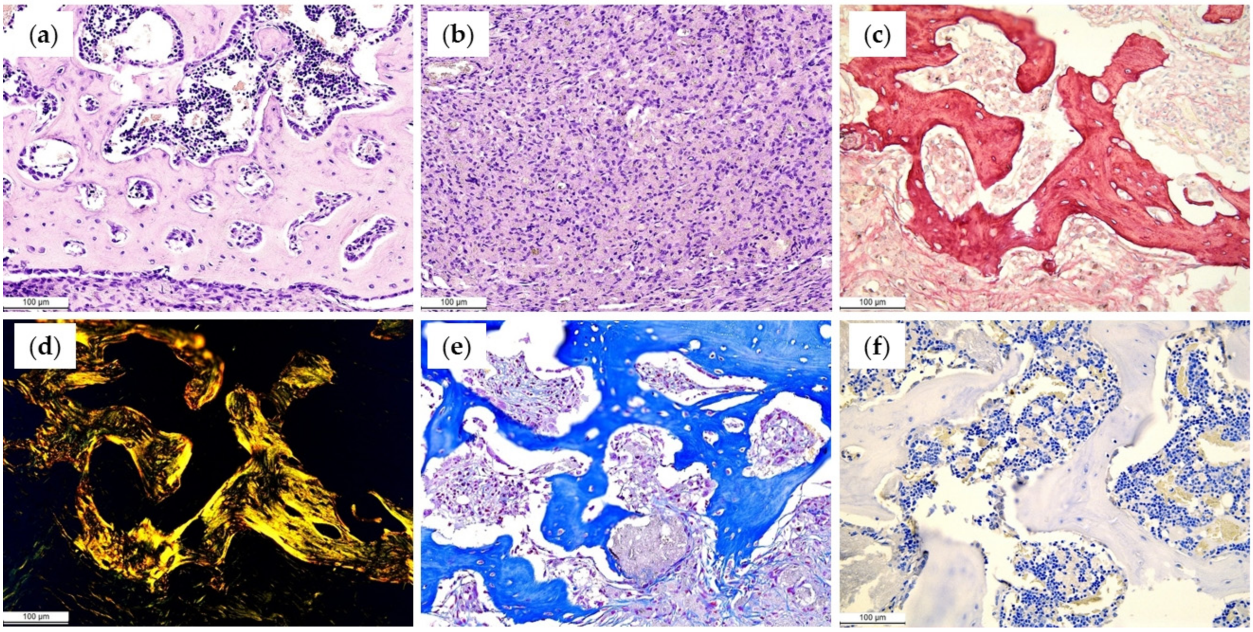

Figure S35. The areas of C6/pCAp-Ce2 implantation 1 month after the surgery. (**a**, **b**) General view of the defect area with bone regeneration (light-field microscopy, hematoxylin-eosin staining, magnification 200×); (**c**) The trabeculae of the bone regenerate turn red (light-field microscopy, picrosirius red staining, magnification 200×); (**d**) Bright anisotropy of the yellow-red color of young bone tissue, the ordered arrangement of collagen fibers and Haversian canals are clearly visible (polarization microscopy, picrosirius red staining, magnification 200×); (**e**) The young bone is uniformly colored blue (light-field microscopy, Mallory staining, magnification 200×); (**f**) Expression of alkaline phosphatase in osteoblasts (colored brown) on the surface of the trabeculae of bone regenerate (light-field microscopy, immunohistochemical staining for alkaline phosphatase, magnification 200×).

References

1. Nifant'ev, I.E.; Shlyakhtin, A.V.; Bagrov, V.V.; Komarov, P.D.; Kosarev, M.A.; Tavtorkin, A.N.; Minyaev, M.E.; Roznyatovsky, V.A.; Ivchenko, P.V. Controlled ring-opening polymerisation of cyclic phosphates, phosphonates and phosphoramidates catalysed by hereroleptic BHT-alkoxy magnesium complexes. *Polym. Chem.* **2017**, *8*, 6806–6816.
2. Nifant'ev, I.E.; Shlyakhtin, A.V.; Bagrov, V.V.; Komarov, P.D.; Tavtorkin, A.N.; Minyaev, M.E.; Kosarev, M.A.; Ivchenko, P.V. Synthesis in aqueous media of poly(ethylene phosphoric acids) by mild thermolysis of homopolymers and block copolymers based on tert-butyl ethylene phosphate. *Eur. Polym. J.* **2018**, *106*, 249–256.
3. Nifant'ev, I.; Tavtorkin, A.; Komarov, P.; Kretov, E.; Korchagina, S.; Chinova, M.; Gavrilov, D.; Ivchenko, P. Dispersant and Protective Roles of Amphiphilic Poly(ethylene phosphate) Block Copolymers in Polyester/Bone Mineral Composites. *Int. J. Mol. Sci.* **2023**, *24*, 11175.
4. Lozhkin, B.; Shlyakhtin, A.; Bagrov, V.; Ivchenko, P.; Nifant'ev, I. Effective stereoselective approach to substituted 1,4-dioxan-2,5-diones as prospective substrates for ring-opening polymerization. *Mendeleev Commun.* **2018**, *28*, 61–63.
5. Shlyakhtin, A.V.; Ryabova, A.V.; Kretov, E.A.; Koroleva, E.A.; Tavtorkin, A.N.; Nifant'ev, I.E.; Bagrov, V.V.; Ivchenko, P.V. Highly statistical PLGAs based on *L*-methylglycolide: the impact of copolymer microstructure on hydrolytic degradation monitored by pH fluorescent sensor. *Eur. Polym. J.* **2025**, *239*, 114309.
